# Metabolic profiling of polycystic ovary syndrome reveals interactions with abdominal obesity

**DOI:** 10.1038/ijo.2017.126

**Published:** 2017-06-27

**Authors:** A Couto Alves, B Valcarcel, V-P Mäkinen, L Morin-Papunen, S Sebert, A J Kangas, P Soininen, S Das, M De Iorio, L Coin, M Ala-Korpela, M-R Järvelin, S Franks

**Affiliations:** 1 Department of Epidemiology and Biostatistics, MRC Health Protection Agency (HPE) Centre for Environment and Health, School of Public Health, Imperial College London, London, UK; 2 Rheumatology Unit, Institute of Child Health, University College London, London, UK; 3 South Australian Health and Medical Research Center, Adelaide, Australia; 4 SAHMRI, School of Biological Sciences, University of Adelaide, Adelaide, Australia; 5 Computational Medicine, Center for Life-Course Health Research, University of Oulu and Oulu University Hospital, Oulu, Finland; 6 Department of Obstetrics and Gynecology, University Hospital of Oulu, Medical Research Center Oulu and PEDEGO Research Unit, University of Oulu, Oulu, Finland; 7 Center for Life-Course Health Research, Northern Finland Cohort Center, Faculty of Medicine, University of Oulu, Oulu, Finland; 8 Biocenter Oulu, University of Oulu, Oulu, Finland; 9 NMR Metabolomics Laboratory, School of Pharmacy, University of Eastern Finland, Kuopio, Finland; 10 Department of Statistical Science, University College London, London, UK; 11 Computational Medicine, School of Social and Community Medicine and the Medical Research Council Integrative Epidemiology Unit, University of Bristol, Bristol, UK; 12 Unit of Primary Care, Oulu University Hospital, Oulu, Finland; 13 Institute of Reproductive and Developmental Biology, Imperial College London, London, UK

## Abstract

**Background:**

Polycystic ovary syndrome (PCOS) is a common reproductive disorder associated with metabolic disturbances including obesity, insulin resistance and diabetes mellitus. Here we investigate whether changes in the metabolic profile of PCOS women are driven by increased tendency to obesity or are specific features of PCOS related to increased testosterone levels.

**Design and methods:**

We conducted an NMR metabolomics association study of PCOS cases (*n*=145) and controls (*n*=687) nested in a population-based birth cohort (*n*=3127). Subjects were 31 years old at examination. The main analyses were adjusted for waist circumference (WC) as a proxy measure of central obesity. Subsequently, metabolite concentrations were compared between cases and controls within pre-defined WC strata. In each stratum, additional metabolomics association analyses with testosterone levels were conducted separately among cases and controls.

**Results:**

Overall, women with PCOS showed more adverse metabolite profiles than the controls. Four lipid fractions in different subclasses of very low density lipoprotein (VLDL) were associated with PCOS, after adjusting for WC and correction for multiple testing (*P*<0.002). In stratified analysis the PCOS women within large WC strata (⩾98 cm) had significantly lower high density lipoprotein (HDL) levels, Apo A1 and albumin values compared with the controls. Testosterone levels were significantly associated with VLDL and serum lipids in PCOS cases with large WC but not in the controls. The higher testosterone levels, adjusted for WC, associated adversely with insulin levels and HOMA IR in cases but not in the controls.

**Conclusions:**

Our findings show that both abdominal obesity and hyperandrogenism contribute to the dyslipidaemia and other metabolic traits of PCOS which all may negatively contribute to the long-term health of women with PCOS.

## Introduction

Polycystic ovary syndrome (PCOS) is a common endocrinopathy in women that affects both reproductive and metabolic function.^1^ PCOS is characterised by metabolic disturbances, particularly insulin resistance and other features of metabolic syndrome that translate into an increased risk of developing type 2 diabetes and cardiovascular disease.

We have previously shown that women with symptoms of PCOS in an unselected general population of northern Finland^2^ have endocrine and metabolic indices which are characteristic of patient populations with clinically diagnosed PCOS and suggest increased cardiovascular disease risk.^3, 4^ However, women with PCOS who have either hyperandrogenism without cycle disturbances or anovulation but with normal androgens levels, are less likely to have metabolic abnormalities.^5^ Although there is evidence that women with PCOS have increased tendency to obesity and abnormalities of lipids and lipoproteins,^4, 6^ there is still controversy^5, 7^ about whether the adverse metabolic indices are mainly related to the syndrome itself or whether they are simply a reflection of obesity and/or abdominal fat distribution.

Comprehensive approaches to gain insights into metabolic variation and diseases, by more refined metabolic phenotyping, have become increasingly popular.^8, 9, 10, 11, 12, 13^ Recent metabolic profiling studies have discovered novel molecular markers associated with the risk for diabetes^14, 15^ and cardiovascular disease.^16, 17, 18^ Metabolomics is increasingly applied to study complex diseases and the relatively new serum nuclear magnetic resonance (NMR) metabolomics platform provides a powerful tool for high-throughput quantitative metabolic profiling.^19, 20, 21^

Here, we present the application of quantitative serum metabolomics to study an extensive sample of a population-based cohort of women with PCOS, and an age-matched reference population. We investigated the metabolic profile of PCOS to determine the impact of abdominal obesity, a feature associated with adverse metabolic function and a common finding in women with PCOS, on metabolic and endocrine health. Because hyperandrogenism may contribute to adipocyte dysfunction and dyslipidemia in PCOS we also assessed whether the adverse changes in the metabolic profile were associated with testosterone levels. Finally, we analysed whether testosterone levels were associated with fasting insulin and insulin resistance, as measured by the homoeostatic model assessment (HOMA IR). The combination of a large, carefully designed population sample, state-of-the art metabolic profiling and extensive laboratory and phenotypic database enabled us specifically to interrogate the complex relationships between PCOS and its metabolic co-morbidities. We were therefore able to relate metabolic indices not only to abdominal obesity but also, independently, to the degree of androgen excess.

## Materials and methods

### Study population

The whole general population-based study comprises 3127 women of the Northern Finland Birth Cohort 1966 who were studied at the age of 31 years. Blood samples were drawn after overnight fasting and serum samples were stored at −80 °C until testosterone and metabolite concentration analyses (methods described previously^22^). Samples were not specifically timed to any phase in the menstrual cycle. We excluded pregnant women, subjects with type 1 or type 2 diabetes, those on oral contraceptives, insulin-sensitising drugs, and on lipid lowering and anti-hypertensive treatments. From all eligible women for the present study 12% were excluded while on contraceptives, further 4% due to other above reasons. All subjects provided written informed consent. The Ethical Committee of the Northern Ostrobothnia Hospital District (Finland) approved the study.

### Clinical and biochemical evaluation of the study population

From among the whole population the women with symptoms of PCOS who met the NICHD (National Institute for Health) diagnostic criteria for PCOS were included in the case group.^23^ Overall, after above exclusions, 145 women were diagnosed with PCOS according to the following criteria: the presence of oligo- or amenorrhoea *and* hyperandrogenism (clinical, that is, hirsutism and/or biochemical). The reference, control, group (687 women) did not present any of these features and was age-matched by birth cohort design. Biochemical hyperandrogenism was defined as serum testosterone concentration >2.4 nmol l^−1^. The cut-off value was derived as previously described^3, 24^ and corresponds to the 75% quartile of the distribution of testosterone values in the reference population.^4^ We defined three strata of waist circumference (WC⩽87 cm, 87<WC<98 and WC⩾98), which were chosen according to well-established criteria derived from a large prospective population study on all-cause mortality.^25^

### Metabolite quantification

The metabolic data were acquired using a high-throughput serum NMR metabolomics platform. The quantified metabolites include 138 measures related to lipoprotein subclasses, serum proteins, lipids, fatty acids and abundant low molecular-weight metabolites, including glycolysis substrates, amino acids, ketone bodies and other small molecules, as listed in Supplementary Table 1. Details of the experimental protocols including sample preparation and spectroscopy have been described previously.^19, 20, 21^

### Statistical analysis

We conducted an overall metabolomics association analysis using logistic regression with PCOS as outcome, with and without (not shown) adjusting for WC as a good proxy for central obesity (in these analyses metabolite measurements were standardised to unit variance). We then stratified the data set based on WC. In each WC stratum, we applied a Welch *t*-test to estimate the metabolite concentration differences between cases and controls complemented with a two-way ANOVA for detecting interactions across strata. In addition, we conducted a linear regression analysis separately in cases and controls, adjusting for WC in each strata to assess differences in the metabolite associations with testosterone. The model fit to each WC strata in cases and controls separately was: testosterone=metabolite+WC. We stratify and correct for WC to avoid residual confounding. A Student’s *t*-test was applied to the normally distributed test-statistic (*β*_pcos_−*β*_control_)/sqrt(s.e._pcos_^2^+s.e._control_^2^) to assess the statistical significance of the regression coefficient difference between cases and controls. We corrected for multiple hypothesis testing with the Bonferroni method by adjusting for the number of independent tests estimated with the Li and Ji method^26^ based on principal component analysis (PCA).^27^ The *P*-value <0.002 was considered as statistically significant. We report the relative difference in the regression coefficient or the relative difference in the mean concentration level as (*X*−*X*_reference_)/*X*_reference_. We assessed the impact of potential confounding by smoking, alcohol consumption and social-economic status which showed that >95% of the PCOS associations with lipoproteins, low molecular weight metabolites and lipids were not significantly affected (<10% of their original regression coefficient) by adding smoking, alcohol or socio-economic status as covariates (Supplementary Table 2). Consequently, we used a reduced model adjusting for or statifying by WC in this study. All analyses were conducted with the R statistical package.^28^

## Results

### Clinical characteristics

In total, 145 women with PCOS by the NICHD diagnostic criteria and their 687 controls were included in the metabolomic analysis. By definition, all cases had oligo- or amenorrheoa and clinical and/or biochemical evidence of androgen excess. 104 cases (77%) had raised serum testosterone, including all those women who did not report being affected by hirsutism. Of those who complained of hirsutism, 53% also had raised serum testosterone. Table 1 shows a comparison of the clinical and biochemical characteristics of women with and without PCOS depicting typical metabolic and endocrine characteristics such as dyslipidaemia, increased insulin resistance, poorer beta cell function, higher diastolic and systolic blood pressure (SBP, DBP), higher luteinizing hormone (LH) and lower sex hormone binding globulin (SHBG) in women with PCOS.

### Overall metabolomics association analyses

**Figure 1:**
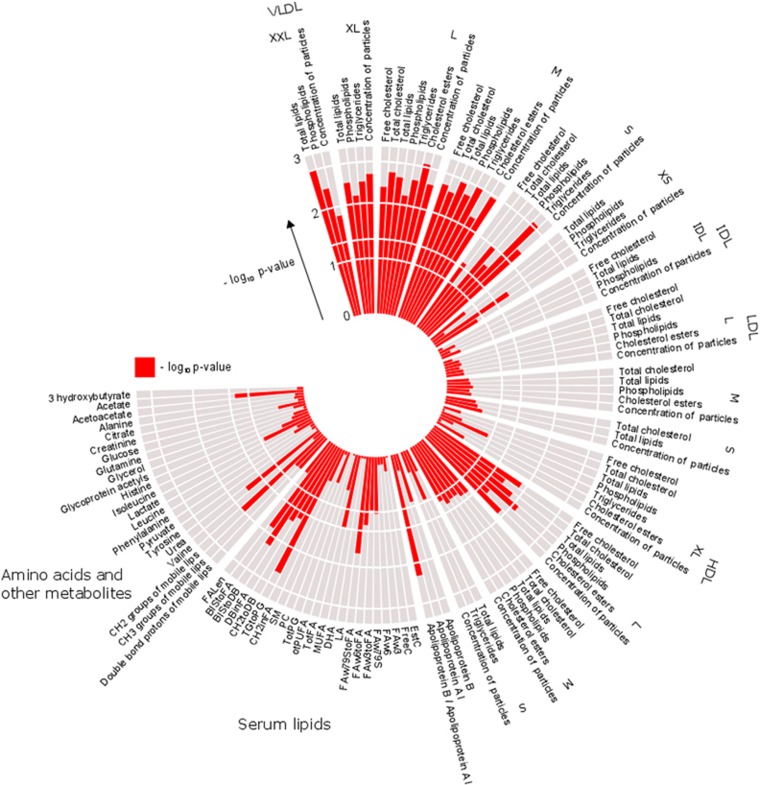
Metabolome-wide pattern of associations with PCOS compared with controls adjusted for waist circumference (−log_10_ *P*-values). Four metabolites in four different VLDL subclasses are significantly associated with PCOS (Bonferroni-corrected significance level α=0.002) independently of WC. Multiple metabolites characterising the larger VLDL and intermediate HDL subclasses showed a tendency (*P*<0.05) for association with PCOS. The red bars denote the −log10 *P*-value of each metabolite, a longer bar indicates a lower *P*-value and therefore higher significance.

In order to further characterise the metabolic profile of PCOS women compared with the controls, we conducted an association analysis of lipoprotein subclasses, lipids and low molecular weight metabolites. Figure 1 shows the metabolome-wide pattern of these associations adjusted for WC in order to report the estimates independent of central obesity. Women with PCOS showed several differences from the reference population in the metabolic profile of the VLDL subclasses. Four lipid measures in four different subclasses of VLDL were associated with PCOS at *P*<0.002 (PCA-based Bonferroni-corrected *P*-value; Figure 1). In addition, seven lipid measures in three different VLDL subclasses showed a suggestive association (*P*<0.004).

**Figure 2:**
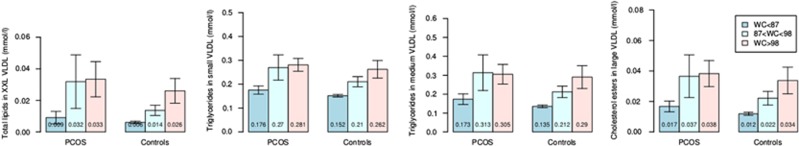
Metabolite profiles in PCOS and control women stratified by WC, illustrating the four metabolites that showed the most significant (*P*<0.002) associations with PCOS that are independent of WC, as identified using logistic regression analysis.

Supplementary Tables 3–5 show detailed association analyses of lipoproteins, lipids and low molecular weight metabolites with PCOS. Overall, triglyceride metabolism was associated with PCOS mainly via larger VLDL particles and total triglycerides levels. Supplementary Table 3 shows that particularly, the total amount of lipids in extremely large VLDL (XXL subclass in Figure 1), the levels of triglycerides in medium VLDL subclasses, cholesterol esters in large VLDL and triglycerides in small VLDL were positively associated with PCOS (odds ratio, OR>2.08 by SD unit increase in metabolite level, *P*<2 × 10^−3^). The total amount of lipids, total cholesterol and the total concentration of cholesterol in medium and large VLDL particles, as well as the phospholipids and free cholesterol in medium VLDL and the triglycerides in large and very large VLDL were suggestively (*P*<0.004) associated with increased risk of PCOS (Supplementary Table 3). However, none of the fatty acids, amino acids or low molecular weight metabolites in serum showed statistically significant association with PCOS in these overall central obesity-adjusted analyses (Supplementary Tables 4 and 5). Analyses were repeated after adjusting for alcohol consumption, smoking and socio-economic status but results did not change substantially. A subset of data, illustrating and comparing metabolite profiles of PCOS women and controls by different strata of WC, is shown in Figure 2. Here, all 4 significant metabolites were associated with PCOS, idependently of WC, in the logistic regression analysis.

### Analysis of metabolites stratified by waist circumference

**Figure 3:**
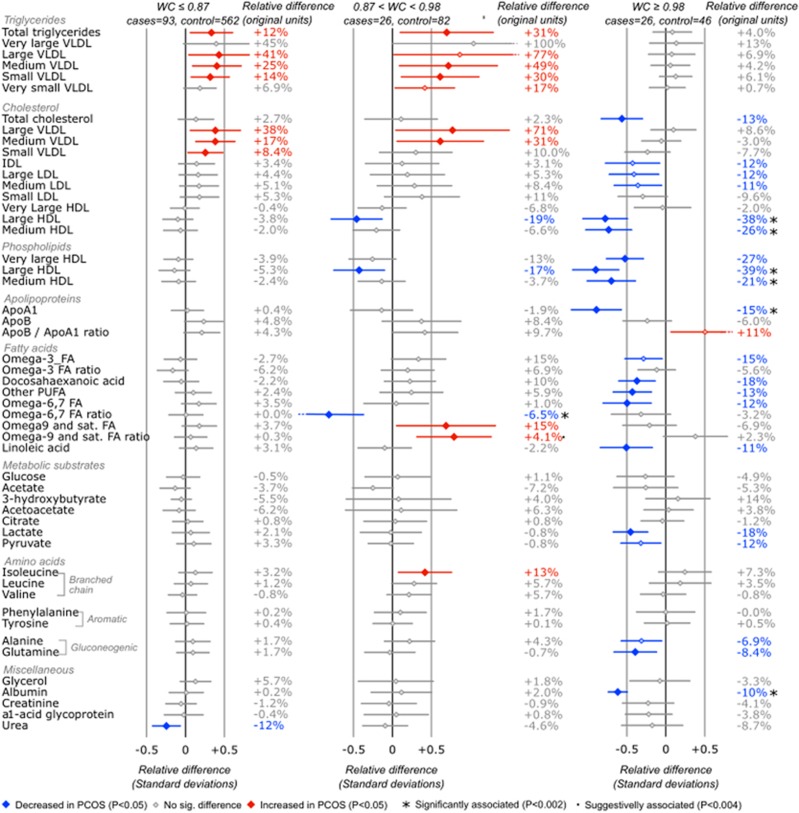
Mean serum metabolite difference between PCOS cases and controls by three waist circumference (WC) strata (⩽87, 87<WC<98, WC⩾98 cm). Overall there are 18 metabolites significantly associated with PCOS in women with large waist circumference (only 8 of which are plotted here, see Supplementary Tables 6–7 for full results), while there were no significant differences in lean women. The points and lines indicate the mean differences with their 95% confidence intervals between cases and controls in standard deviations. The relative difference was calculated from the original data without scaling as [mean(cases)−mean(controls)]/mean(controls). Significant and suggestive differences after multiple testing correction indicated with specific characters. Serum metabolites levels that are higher in PCOS cases are in red and those that are lower in blue. Omega 3 FA ratio, omega 6,7 FA ratio and omega-9 FA ratio denote the omega metabolite ratio to total fatty acids. Apo denotes apolipoprotein; FA, fatty acid; HDL, high density lipoprotein; IDL, intermediate density lipoprotein; LDL, low density lipoprotein; TG, triglycerides; VLDL, very low density lipoprotein.

To further understand the interaction between body fat distribution and biochemical data in PCOS women, we conducted a stratified analysis of metabolites by the pre-ordained tertiles of WC in both cases and controls. We found 18 metabolites significantly associated with PCOS in women with large waist circumference (WC>98 cm), only one in the middle WC stratum, but none in women with smaller WC (Supplementary Tables 6 and 7). Interestingly, the metabolic ‘impact’ of PCOS was modest in women with a small waist circumference apart from a tendency for higher (*P*<0.05) VLDL cholesterol and triglycerides (WC⩽87 cm) (Figure 3). In the intermediate stratum (87 cm<WC<98 cm) only the ratio of omega 6/7 to total fatty acids was statistically significantly lower in PCOS women than their controls (Supplementary Table 6). In the large waist circumference stratum, the concentration of phospholipids, cholesterol, free cholesterol and cholesterol esters in HDL subclasses were significantly (*P*<0.002) decreased, as well as the diameter and concentration of HDL particles. Albumin concentration and total choline were lower in PCOS women compared with the controls (Supplementary Tables 6 and 7). In the large WC stratum (Figure 3), the concentration of the cholesterol in LDL, fatty acids and gluconeogenic metabolites showed a decreasing tendency (*P*<0.05) in women with PCOS while the ratio of Apo B/Apo A1 showed an increasing tendency (Figure 3). Interestingly, the impact of WC on HDL profile was stronger in women with PCOS than controls, that is, PCOS women had lower HDL values. In particular, the average magnitude of change in HDL lipid profile between small and large WC in PCOS women was 32% as opposed to only 12% in controls (change=100% × |*C*_largeWC_−*C*_lowWC_|/*C*_lowWC_). In the intermediate level of WC, the change was smaller (13 and 7%) but the relative difference between cases and controls was still considerable. There were no statistically significant differences in low molecular weight metabolites between cases and controls by WC strata (data not shown). In conclusion, women with PCOS and abdominal obesity (waist circumference⩾98 cm) had an altered metabolic profile in terms of HDL subclasses, total choline, albumin, apolipoprotein A1 and Apo B/Apo A1 ratio.

### Relationship of metabolic profiles to serum testosterone

We examined (a) whether the differences in concentration levels of HDL, apolipoproteins and other metabolites were related to higher testosterone levels in women with PCOS compared with the controls and (b) how this relationship was affected by central obesity. We therefore conducted a metabolite association analysis with testosterone separately in women with PCOS and controls for all WC strata (also adjusting for WC in strata).

**Figure 4:**
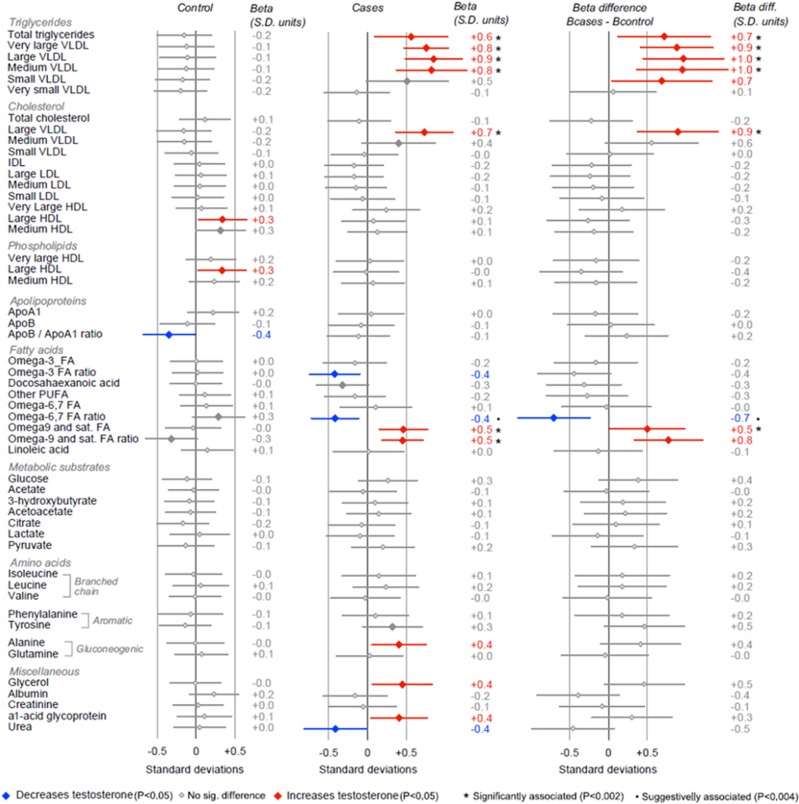
Serum metabolite associations with testosterone levels in PCOS cases and controls with large waist circumference (WC⩾98). Overall there are 30 metabolites significantly associated with testosterone in PCOS but not in control women with large waist circumference (only 6 of these plotted here, see Supplementary Tables 8–10 for full data set), suggesting that androgens levels correlate with adverse metabolic profiles of VLDL and lipid fractions in abdominally obese but not in otherwise lean PCOS women. No associations found in lean or intermediate obese women. The points and horizontal lines indicate the regression coefficient slopes (*β*) for testosterone with their 95% confidence intervals in cases and controls. Betas presented in standard deviation (s.d.) units and the beta difference was calculated as *β*(cases)−*β*(controls) and presented in s.d. units. Significant and suggestive differences, after correction for multiple testing, indicated with specific characters. Serum metabolites levels that are positively associated with testosterone are in red and those that are negatively associated in blue. Omega 3 FA ratio, omega 6,7 FA ratio and omega-9 FA ratio denote the omega metabolite ratio to total fatty acids. Apo denotes apolipoprotein; FA, fatty acid; HDL, high density lipoprotein; IDL, intermediate density lipoprotein; LDL, low density lipoprotein; TG, triglycerides; VLDL, very low density lipoprotein.

We found 8 significant associations of testosterone with lipids and 22 with lipoproteins in PCOS women with large (WC⩾98) but not in women with intermediate and smaller waist circumference (except for one positive association with the ratio of omega 6 and 7 in intermediate strata). The relationship of testosterone with VLDL, and to a lesser extent LDL and HDL profiles, showed significant differences between the cases and controls (Figure 4). The associations for strata of WC⩾98 are shown in Supplementary Tables 8–10.

The lipid profile of PCOS women with abdominal obesity was highly associated with testosterone (Figure 4; Supplementary Table 8). Total triglycerides, the ratio of triglycerides to phosphoglycerides, omega-9 and saturated fatty acids, the ratio of omega-9 and saturated fatty acids to total fatty acids and the average number of methylene groups per double bond showed positive association with levels of testosterone in PCOS women but not in controls (*β*>0.17, *P*<1.9 × 10^−3^). Conversely, the ratio of bisallylic groups to fatty acids, the ratio of bisallylic groups to double bonds as well as the average number of double bonds in fatty acid chains were all negatively associated with levels of testosterone in PCOS women but not in controls (*β*<−4.9, *P*<1.6 × 10^−3^).

The lipoprotein profile of PCOS women with abdominal obesity showed positive group-specific associations with testosterone levels (Supplementary Table 9). In the set of statistically significant associations with testosterone, the difference in regression coefficient between cases and controls remained statistically significant after correction for multiple comparisons for most metabolites. Triglycerides in the larger VLDL subclasses, the serum concentration of VLDL particles, the VLDL mean diameter as well as the phospholipids and total lipids in VLDL subclasses (ranging from medium to extremely large particles), and the total cholesterol, cholesterol esters and free cholesterol in large VLDL subclasses were all positively associated with testosterone levels (*β*>0.73, *P*<1.4 × 10^−3^). Conversely, the mean diameter of LDL particles showed a negative association with testosterone levels in PCOS women but not in controls (*β*=−3.4, *P*=3.9 × 10^−4^). We did not find significant associations of testosterone with low molecular weight metabolites in overall analyses or by WC strata (Supplementary Table 10).

### Testosterone relationship to insulin resistance

In order to investigate further the role on testosterone on the glycaemic status in PCOS we conducted a regression analysis of fasting insulin levels and an index of insulin resistance (HOMA IR) (Table 2) adjusting for WC.

Testosterone levels were associated with insulin levels in cases (*β*=0.71, *P*=0.047) but not in controls (*β*=0.003, *P*=0.99). There was a suggestion that the impact of testosterone on insulin levels was stronger (*P*=0.096) in PCOS, indicating possible differences in the relationship between insulin and testosterone between PCOS and the reference group.

Testosterone was also significantly associated with insulin resistance (HOMA IR) in cases (*β*=0.092, *P*=0.042) but not in controls (*β*=0.001, *P*=0.97). Interestingly, the association of WC with insulin levels or resistance was significantly stronger in cases (*P*<3.7 × 10^−5^) than in the controls.

## Discussion

Metabolic abnormalities are a common feature of women with PCOS but there remains controversy about whether such abnormalities, in particular dyslipidemia, are a function of PCOS itself or of accompanying obesity, and, in particular abdominal obesity which is a well-established risk factor for metabolic and cardiovascular disease. We reasoned that since PCOS itself is associated with increased risk factors for cardiovascular disease, the impact of PCOS diagnosis, over and above the effect of obesity, would compound that risk. This study sought to address this question by a comprehensive characterisation of the metabolic profile of PCOS women using, for the first time in this context, the serum concentrations of metabolites analysed by state of the art quantitative NMR metabolomics platform in a large cohort of women who met the NICHD diagnostic criteria for PCOS. We conducted both WC stratified and adjusted analyses and assessed the impact of testosterone levels on the metabolic profile of PCOS and control women over a range of WC. We found a significant interaction between obesity, testosterone and dyslipidemia in women with PCOS, which indicates that the syndrome itself is an important player in the lipoprotein abnormalities beyond the effects of obesity *per se*.

### Association analysis

Previous studies have shown that dyslipidemia is common in PCOS.^29, 30^ In particular, triglycerides and LDL cholesterol have been found to be higher in women with PCOS, while HDL-C was lower. Metabolomic studies have previously been performed in women with PCOS^31, 32, 33, 34^ but with the exception of that by Zhao *et al.* (2012), the numbers of PCOS subjects involved have been small and no other study has included a large, matched reference population or stratified the results systematically to account for the effects of abdominal obesity and serum testosterone.

In our study, adjusting for WC and using a state of the art metabolomics platform, the lipoprotein profile showed independent associations of various VLDL subclasses with PCOS. After correction for multi-comparison, triglycerides in small and medium VLDL subclasses were positively associated with PCOS, which is consistent with the literature that shows an overall increase of TG levels in VLDL.^35^ We also found that cholesterol esters in large VLDL and the total amount of lipids in extremely large VLDL (which are new markers that were measured using the metabolomics platform) were increased in PCOS. Lipid content in VLDL subclasses, which has previously been shown to be associated with obesity, was investigated in this study using a stratified analysis. Interestingly, although we analysed 14 lipoprotein subclasses characterising all major fractions (VLDL, IDL, LDL and HDL), only lipid measures in larger VLDL subclasses were affected in women with PCOS.

### Stratified analysis

Previous studies^36, 37^ have found that both lean and obese women show signs of insulin resistance and reduced levels of cholesterol in HDL, HDL_2_ and HDL_3_ subclasses. PCOS women, as opposed to controls, have shown significantly decreased insulin sensitivity with increasing BMI^36^ and low-grade chronic inflammation.^38^

In the present study, using a stratified analysis by WC, we found decreased HDL cholesterol in PCOS women with abdominal obesity, which is consistent with results of previous studies^4, 39, 40, 41^ in which stratification by BMI was used. We also found new biochemical markers of PCOS in women with abdominal obesity, including albumin and total choline. In a recent study albumin levels have been associated with increased total mortality.^42^ Importantly, our results show that the lipid profile of HDL subclasses is significantly more influenced by the degree of abdominal obesity in PCOS women than in controls. Phospholipids, total cholesterol, free cholesterol, cholesterol esters and total lipids in the larger HDL subclasses were lower in PCOS. Consistent with these results, the concentration and the mean diameter of HDL particles were smaller in PCOS women with abdominal obesity. These changes in HDL concentration and HDL particle size are known to be associated with coronary artery disease^43^ and may increase susceptibility to coronary events.^44^

Importantly, Apo A1 concentrations in PCOS women with abdominal obesity were significantly lower than in controls, which is also shown to be accompanied by lower HDL levels. Previous studies^39^ in obese and overweight women (BMI>27) did not find a significant difference, possibly due to smaller sample size or due to stratification by BMI instead of WC. Our association analysis results were consistent with this finding, showing that the ratio of Apo B and A1 was positively associated with PCOS. The multivariate pattern of decreased Apo A1, HDL-C and HDL-phospholipids have been previously linked to inflammation-associated dyslipidaemia in obesity.^45^ This is corroborated by previous cross-sectional studies that found elevated CRP levels and increased low-grade chronic inflammation in PCOS women.^46^ These results suggest that PCOS women may indeed be at higher risk of cardiovascular diseases such as atherosclerosis. To date, we lack secure longitudinal data on cardiovascular events in women with PCOS but the data here support the view that PCOS women who carry these risk factors may require careful clinical monitoring from an early age.^47^

### Relationship of metabolites to serum testosterone levels

Previous interventional studies showed that intramuscular injections of testosterone esters in young females undergoing gender reassignment increased visceral fat^48^ and resulted in insulin resistance^49^ and so did the administration of oral methyltestosterone to healthy pre-menopausal women.^50^ Mirroring these observations in adolescent girls with PCOS, metabolic syndrome risk increased 4-fold for every quartile increase in testosterone after adjusting for SHBG.^51^ The application of subcutaneous testosterone implants in women with pre-menstrual syndrome decreased Apo A1 and HDL-C and increased VLDL-C, while in pre-menopausal obese women, the same treatment showed a small decrease of serum HDL-C and TC.^52^ In contrast, in PCOS women, the free androgen index (Testosterone/SHBG) was positively correlated with TC, TG, LDL-C, Apo B and phospholipids (PL) (although after adjusting for age and BMI the association was not significant).

In our study, PCOS women with abdominal obesity showed differences in the lipid and lipoprotein profiles in relation to serum testosterone. In these women, testosterone levels were positively associated with serum TG as well as with PL, TG and TC in larger VLDL subclasses, which is consistent with other reported studies in women with PCOS. However, we did not find the negative associations of testosterone with Apo A1 and HDL-C that have been observed in interventional studies of testosterone treatment. This suggests that the impact of testosterone on lipid metabolism in PCOS differs from control women with normal pre-treatment androgen levels. We also found that total lipids in VLDL subclasses were positively correlated with testosterone, the concentrations and mean diameters of larger VLDL particles were also positively correlated with testosterone, while the mean diameter of LDL particles was negatively correlated. Interestingly, omega-9 and saturated fatty acids were also positively correlated with testosterone. These novel markers may have public health significance as larger VLDL particle size is associated with type 1 diabetes in women^53^ and PCOS women are recommended a diet with increased levels of omega-9 (a monounsaturated fatty acid—MUFA).^54^ The rise of VLDL and decrease of HDL associated with high testosterone levels potentially increase cardiovascular disease risk. These metabolic characteristics are exacerbated in women with PCOS and abdominal obesity (WC⩾0.98) suggesting an interaction between testosterone and abdominal obesity that putatively affect metabolic risk factors of cardiovascular disease. The converse is that this relationship is not observed in women with PCOS who have normal WC which is important in counselling women witth PCOS about metabolic risk. Taken together, these results suggest that high testosterone levels adversely affect a range of metabolites, modulate the risk for metabolic syndrome, diabetes and cardiovascular disease and have an important role in the pathophysiology of PCOS. Our results are consistent with the conclusions of the recent study by Dumesic and colleagues that hyperandrogenism, even in lean women with PCOS, is associated with preferential deposition of intraabdominal fat that in turn results in impaired storage of fat in subcutaneous adipocytes and consequent metabolic dysfunction.^55^ It is conceivable that the dyslipidemia that we observe in women with PCOS is explained, at least in part, by the impact of excess androgens on the ability of adipocytes to safely store excess fat.

### Strengths and limitations

A major strengths of the study are the systematic stratification and adjustment of analyses for WC as a measure of abdominal obesity and the homogeneity of the study populaton. Both cases and their controls were retrived from the NFBC1966, extremely well characterised general population sample. This eliminates, as far as possible, the bias often encountered in these studies. Blood samples were not taken at the same stage of the cycle in women with regular menses. This might conceivably introduce some bias in interpretation of lipid levels but the available data are inconsistent and any such cyclical variation is small and most obvious in the preovulatory phase.^56^ But effectively the sampling was randomly performed, thus minimising any possible effect of cyclical changes. Although cases with PCOS were initially selected on the basis of self-reported symptoms, we only included subjects in the study group who met the criteria for the NIHCD definition of the syndrome. All PCOS women included here suffered from infrequent or absent periods and had clinical and/or biochemical evidence of hyperandrogenism. Significantly, 77% of women complaining of hirsutism had a raised serum testosterone level. Furthermore, we have shown, in previous studies, that the biochemical characteristics of those women with self-reported symptoms of PCOS are very similar to clinic-based patients with PCOS.^2^ That is particularly the case in those women in the cohort who have both oligomenorrhea and hyperandrogenism. We recognise that HOMA IR is not an ideal index of insulin resistance but in general population studies it is often impractical to perform euglycemic hyperinsulinemic clamp studies in all subjects and, in this case, HOMA IR and fasting insulin are satisfactory surrogate measures.

## Conclusions

**Figure 5:**
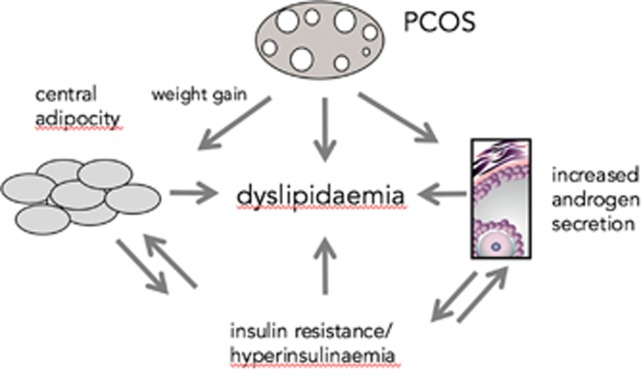
Concept diagram illustrating the mutlifactorial basis of dyslipidemia in women with PCOS. PCOS, hyperandogenism and abdominal adiposity (and associated insulin resistance) independently and in combination are related to lipid metabolism.

In conclusion, we have found metabolic abnormalities, in particular, alterations in lipid metabolism, in women with PCOS which persist after correction for central body adiposity. Alterations in HDL lipid profile, Apo A1 and albumin levels were most significant in those women within the stratum of highest waist circumference whereas those without central obesity were very similar to the reference population. Our results support the view that PCOS, hyperandrogenism and abdominal obesity act independently but also, crucially, in combination, to affect lipid metabolism (Figure 5). The importance of our results are further strengthened by a report from Women’s Health Study from the USA that emphasizes the role of serum HDL-C levels in the risk of cardiovascular events.^57^ Our findings have therefore important implications for management of long-term health in women with PCOS. Abdominal obesity itself carries an increased risk of metabolic dysfunction and its associated pathology; increasing waist cicumference in women with PCOS hightens those risks. PCOS has only small effect on the metabolic profile in lean women but the importance of dietary advice and healthy eating is particularly clear in women with both PCOS and abdominal obesity.

## Figures and Tables

**Table 1:** Clinical and biochemical characteristics of women with PCOS by the NICHD criteria^a^ and their controls

	*Cases,* n=*145*				*Controls,* n=*687*				P*-value*
	*95% CI*				*95% CI*				
	*Mean*	*Lower*	*Upper*	*s.d.*	*Mean*	*Lower*	*Upper*	*s.d.*	
Waist circumference (cm)	84.9	82.3	87.5	16.5	78	77.2	78.9	11.2	7.4E−6
Waist-hip ratio	0.84	0.83	0.85	0.08	0.8	0.79	0.81	0.1	9.2E−6
BMI (kg m^−^^2^)	26.5	25.5	27.6	6.6	23.7	23.4	24.1	4.4	9.1E−6
HOMA insulin resistance (IR)	1.25	1.13	1.37	0.73	0.98	0.95	1.01	0.41	3.5E−5
HOMA beta cell function	108.7	103	114.4	35.6	97.5	95.7	99.3	24.6	5.5E−4
HOMA insulin sensitivity	98.5	92.2	104.9	39.8	114.7	112	117.4	36.7	2.3E−5
Glucose (mmol l^−1^)	5.0	4.9	5.1	0.4	4.9	4.86	4.94	0.5	1.8E−2
Insulin (mU l^−1^)	9.7	8.8	10.6	5.8	7.6	7.4	7.8	3.2	3.7E−5
SHBG (nmol l^−1^)	56.5	49.7	63.3	42	65.9	63.4	68.4	33.5	1.5E−2
Testosterone (nmol l^−1^)	2.9	2.7	3.0	1.0	1.6	1.57	1.63	0.4	1.2E−34
Luteinizing hormone (U l^−1^)	10.8	7.7	13.8	18.8	6.1	5.6	6.6	6.9	4.9E−3
Systolic blood pressure (mmHg)	122.0	119.2	124.5	13.8	118.6	117.7	119.5	11.7	6.0E−3
Diastolic blood pressure (mmHg)	77.1	75.6	79.5	11.7	74	73.2	74.8	10.3	1.0E−3
Total cholesterol (mmol l^−1^)	5.0	4.8	5.2	1.0	4.8	4.7	4.9	0.9	1.1E−2
LDL cholesterol (mmol l^−1^)	3.0	2.8	3.1	0.9	2.75	2.7	2.8	0.9	1.2E−2
HDL cholesterol (mmol l^−1^)	1.5	1.5	1.6	0.4	1.7	1.67	1.73	0.4	8.9E−4
Triglycerides (mmol l^−1^)	1.1	1.0	1.2	0.6	0.86	0.83	0.89	0.4	8.1E−6

Abbreviations: HDL, high density lipoprotein; HOMA, homoeostatic model assessment; LDL, low density lipoprotein; SHBG, sex hormone binding globulin.

a By National Institute for Health, USA.

**Table 2:** The relationship of fasting insulin and insulin resistance (HOMA IR) with testosterone levels

*Outcome*	*Exposure*	*Cases*			*Controls*			*Beta difference*
		β	*s.e.*	P*-value*	β	*s.e.*	P*-value*	P*-value*
Insulin (mU l^−1^)	Testosterone (nmol l^−1^)	0.712	0.354	4.70E−02	0.003	0.236	9.90E−01	9.6E−02^·^
(Model 1)	WC (cm)	0.242	0.021	2.00E−16	0.147	0.009	2.00E−16	2.80E−05
HOMA IR	Testosterone (nmol l^−1^)	0.092	0.045	4.20E−02	0.001	0.031	9.70E−01	9.5E−02^·^
(Model 2)	WC (cm)	0.031	0.003	2.00E−16	0.019	0.001	2.00E−16	3.70E−05

Abbreviation: HOMA, homoeostatic model assessment.

Model 1: Insulin=Testosterone+WC; Model 2: HOMA IR=Testosterone+WC; Beta values for insulin and HOMA IR levels are mutually adjusted^a^ and by unit increase in exposure level.

a The association between testosterone and outcomes are adjusted for waist circumference (WC) and between WC and outcome by testosterone.

## References

[bib1] Franks S. Polycystic ovary syndrome. N Engl J Med 1995; 333: 853–861. 7651477 10.1056/NEJM199509283331307

[bib2] Taponen S, Ahonkallio S, Martikainen H, Koivunen R, Ruokonen A, Sovio U et al. Prevalence of polycystic ovaries in women with self reported symptoms of oligomenorrhoea and/or hirsutism: Northern Finland Birth Cohort 1966 Study. Hum Reprod 2004; 19: 1083–1088. 15044401 10.1093/humrep/deh214

[bib3] Taponen S, Martikainen H, Järvelin M-R, Laitinen J, Pouta A, Hartikainen A-L et al. Hormonal profile of women with self-reported symptoms of oligomenorrhea and/or hirsutism: Northern Finland Birth Cohort 1966 Study. J Clin Endocrinol Metab 2003; 88: 141–147. 12519843 10.1210/jc.2002-020982

[bib4] Taponen S, Martikainen H, Järvelin M-R, Sovio U, Laitinen J, Pouta A et al. Metabolic cardiovascular disease risk factors in women with self-reported symptoms of oligomenorrhea and/or hirsutism: Northern Finland Birth Cohort 1966 Study. J Clin Endocrinol Metab 2004; 89: 2114–2118. 15126528 10.1210/jc.2003-031720

[bib5] Barber TM, Wass JAH, McCarthy MI, Franks S. Metabolic characteristics of women with polycystic ovaries and oligo-amenorrhoea but normal androgen levels: implications for the management of polycystic ovary syndrome. Clin Endocrinol 2007; 66: 513–517. 10.1111/j.1365-2265.2007.02764.x 17371468

[bib6] Barber TM, McCarthy MI, Wass JAH, Franks S. Obesity and polycystic ovary syndrome. Clin Endocrinol 2006; 65: 137–145. 10.1111/j.1365-2265.2006.02587.x 16886951

[bib7] Apridonidze T, Essah PA, Iuorno MJ, Nestler JE. Prevalence and characteristics of the metabolic syndrome in women with polycystic ovary syndrome. J Clin Endocrinol Metab 2005; 90: 1929–1935. 15623819 10.1210/jc.2004-1045

[bib8] Ala-Korpela M. Critical evaluation of 1H NMR metabonomics of serum as a methodology for disease risk assessment and diagnostics. Clin Chem Lab Med 2008; 46: 27–42. 18020967 10.1515/CCLM.2008.006

[bib9] Lewis GD, Asnani A, Gerszten RE. Application of metabolomics to cardiovascular biomarker and pathway discovery. J Am Coll Cardiol 2008; 52: 117–123. 18598890 10.1016/j.jacc.2008.03.043 PMC3204897

[bib10] Holmes E, Wilson ID, Nicholson JK. Metabolic phenotyping in health and disease. Cell 2008; 134: 714–717. 18775301 10.1016/j.cell.2008.08.026

[bib11] Ala-Korpela M, Kangas AJ, Soininen P. Quantitative high-throughput metabolomics: a new era in epidemiology and genetics. Genome Med 2012; 4: 36. 22546737 10.1186/gm335 PMC3446264

[bib12] Alves AC, Li JV, Garcia-Perez I, Sands C, Barbas C, Holmes E et al. Characterization of data analysis methods for information recovery from metabolic 1 H NMR spectra using artificial complex mixtures. Metabolomics 2012; 8: 1170–1180.

[bib13] Murri M, Insenser M, Escobar-Morreale HF. Metabolomics in polycystic ovary syndrome. Clin Chim Acta 2014; 429: 181–188. 24368231 10.1016/j.cca.2013.12.018

[bib14] Rhee EP, Cheng S, Larson MG, Walford GA, Lewis GD, McCabe E et al. Lipid profiling identifies a triacylglycerol signature of insulin resistance and improves diabetes prediction in humans. J Clin Invest 2011; 121: 1402–1411. 21403394 10.1172/JCI44442 PMC3069773

[bib15] Wang TJ, Larson MG, Vasan RS, Cheng S, Rhee EP, McCabe E et al. Metabolite profiles and the risk of developing diabetes. Nat Med 2011; 17: 448–453. 21423183 10.1038/nm.2307 PMC3126616

[bib16] Shah SH, Bain JR, Muehlbauer MJ, Stevens RD, Crosslin DR, Haynes C et al. Association of a peripheral blood metabolic profile with coronary artery disease and risk of subsequent cardiovascular events. Circ Cardiovasc Genet 2010; 3: 207–214. 20173117 10.1161/CIRCGENETICS.109.852814

[bib17] Shah SH, Kraus WE, Newgard CB. Metabolomic profiling for the identification of novel biomarkers and mechanisms related to common cardiovascular diseases: form and function. Circulation 2012; 126: 1110–1120. 22927473 10.1161/CIRCULATIONAHA.111.060368 PMC4374548

[bib18] Wurtz P, Makinen VP, Soininen P, Kangas AJ, Tukiainen T, Kettunen J et al. Metabolic signatures of insulin resistance in 7,098 young adults. Diabetes 2012; 61: 1372–1380. 22511205 10.2337/db11-1355 PMC3357275

[bib19] Soininen P, Kangas AJ, Wurtz P, Tukiainen T, Tynkkynen T, Laatikainen R et al. High-throughput serum NMR metabonomics for cost-effective holistic studies on systemic metabolism. Analyst 2009; 134: 1781–1785. 19684899 10.1039/b910205a

[bib20] Inouye M, Kettunen J, Soininen P, Silander K, Ripatti S, Kumpula LS et al. Metabonomic, transcriptomic, and genomic variation of a population cohort. Mol Syst Biol 2010; 6: 441. 21179014 10.1038/msb.2010.93 PMC3018170

[bib21] Kettunen J, Tukiainen T, Sarin AP, Ortega-Alonso A, Tikkanen E, Lyytikainen LP et al. Genome-wide association study identifies multiple loci influencing human serum metabolite levels. Nat Genet 2012; 44: 269–276. 22286219 10.1038/ng.1073 PMC3605033

[bib22] Taponen S, Martikainen H, Järvelin M-R, Laitinen J, Pouta A, Hartikainen A-L et al. Hormonal profile of women with self-reported symptoms of oligomenorrhea and/or hirsutism: Northern Finland Birth Cohort 1966 Study. J Clin Endocrinol Metab 2003; 88: 141–147. 12519843 10.1210/jc.2002-020982

[bib23] Zawadzki J, Dunaif A. Diagnostic criteria for polycystic ovary syndrome: towards a rational approach. In: Dunaif A, Givens J, Haseltine F, Merriam G. (eds). Polycystic Ovary Syndrome. edn. Blackwell Scientific: Boston, 1992; 377–384.

[bib24] Kousta E, Cela E, Lawrence N, Penny A, Millauer B, White D et al. The prevalence of polycystic ovaries in women with a history of gestational diabetes. Clin Endocrinol 2000; 53: 501–507. 10.1046/j.1365-2265.2000.01123.x 11012576

[bib25] Kramer H, Shoham D, McClure LA, Durazo-Arvizu R, Howard G, Judd S et al. Association of waist circumference and body mass index with all-cause mortality in CKD: The REGARDS (Reasons for Geographic and Racial Differences in Stroke) Study. Am J Kidney Dis 2011; 58: 177–185. 21601327 10.1053/j.ajkd.2011.02.390 PMC3144322

[bib26] Li J, Ji L. Adjusting multiple testing in multilocus analyses using the eigenvalues of a correlation matrix. Heredity 2005; 95: 221–227. 16077740 10.1038/sj.hdy.6800717

[bib27] Gao X, Starmer J, Martin ER. A multiple testing correction method for genetic association studies using correlated single nucleotide polymorphisms. Genet Epidemiol 2008; 32: 361–369. 18271029 10.1002/gepi.20310

[bib28] R Core TeamR: A Language and Environment for Statistical Computing. R Foundation for Statistical Computing: Vienna, Austria, 2012.

[bib29] Wild RA, Rizzo M, Clifton S, Carmina E. Lipid levels in polycystic ovary syndrome: systematic review and meta-analysis. Fertil Steril 2011; 95: 1073–1079. e1011. 21247558 10.1016/j.fertnstert.2010.12.027

[bib30] Zhao Y, Fu L, Li R, Wang L-N, Yang Y, Liu N-N, Zhang C-M et al. Metabolic profiles characterizing different phenotypes of polycystic ovary syndrome: plasma metabolomics analysis. BMC Med 2012; 10: 153. 23198915 10.1186/1741-7015-10-153 PMC3599233

[bib31] Dong F, Deng D, Chen H, Cheng W, Li Q, Luo R et al. Serum metabolomics study of polycystic ovary syndrome based on UPLC-QTOF-MS coupled with a pattern recognition approach. Anal Bioanal Chem 2015; 407: 4683–4695. 25860656 10.1007/s00216-015-8670-x

[bib32] Haoula Z, Ravipati S, Stekel DJ, Ortori CA, Hodgman C, Daykin C et al. Lipidomic analysis of plasma samples from women with polycystic ovary syndrome. Metabolomics 2015; 11: 657–666. 25972770 10.1007/s11306-014-0726-y PMC4419155

[bib33] Whigham LD, Butz E, Dashti D, Tonelli HM, Johnson LK, Cook ME et al. Metabolic evidence of diminished lipid oxidation in women with polycystic ovary syndrome. Curr Metabol 2013; 1: 269–278. 10.2174/2213235X01666131203230512 PMC3994884 24765590

[bib34] Zhao X, Xu F, Qi B, Hao S, Li Y, Li Y et al. Serum metabolomics study of polycystic ovary syndrome based on liquid chromatography–mass spectrometry. J Proteome Res 2014; 13: 1101–1111. 24428203 10.1021/pr401130w

[bib35] Wild RA, Painter P, Coulson PB, Carruth KB, Ranney G. Lipoprotein lipid concentrations and cardiovascular risk in women with polycystic ovary syndrome. J Clin Endocrinol Metab 1985; 61: 946–951. 4044782 10.1210/jcem-61-5-946

[bib36] Rajkhowa M, Neary R, Kumpatla P, Game F, Jones P, Obhrai M et al. Altered composition of high density lipoproteins in women with the polycystic ovary syndrome 1. J Clin Endocrinol Metab 1997; 82: 3389–3394. 9329374 10.1210/jcem.82.10.4318

[bib37] Conway GS, Agrawal R, Betteridge D, Jacobs H. Risk factors for coronary artery disease in lean and obese women with the polycystic ovary syndrome. Clin Endocrinol 1992; 37: 119–125. 10.1111/j.1365-2265.1992.tb02295.x 1395062

[bib38] Kelly CC, Lyall H, Petrie JR, Gould GW, Connell JM, Sattar N. Low grade chronic inflammation in women with polycystic ovarian syndrome. J Clin Endocrinol Metab 2001; 86: 2453–2455. 11397838 10.1210/jcem.86.6.7580

[bib39] Rajkhowa M, Neary RH, Kumpatla P, Game FL, Jones PW, Obhrai MS et al. Altered composition of high density lipoproteins in women with the polycystic ovary syndrome. J Clin Endocrinol Metab 1997; 82: 3389–3394. 9329374 10.1210/jcem.82.10.4318

[bib40] Pirwany IR, Fleming R, Greer IA, Packard CJ, Sattar N. Lipids and lipoprotein subfractions in women with PCOS: relationship to metabolic and endocrine parameters. Clin Endocrinol 2001; 54: 447–453. 10.1046/j.1365-2265.2001.01228.x 11318779

[bib41] Phelan N, O'Connor A, Kyaw-Tun T, Correia N, Boran G, Roche HM et al. Lipoprotein subclass patterns in women with polycystic ovary syndrome (PCOS) compared with equally insulin-resistant women without PCOS. J Clin Endocrinol Metab 2010; 95: 3933–3939. 20519354 10.1210/jc.2009-2444

[bib42] Fischer K, Kettunen J, Würtz P, Haller T, Havulinna AS, Kangas AJ et al. Biomarker profiling by nuclear magnetic resonance spectroscopy for the prediction of all-cause mortality: an observational study of 17,345 persons. PLoS Med 2014; 11: e1001606. 24586121 10.1371/journal.pmed.1001606 PMC3934819

[bib43] El Harchaoui K, Arsenault BJ, Franssen R, Després J-P, Hovingh GK, Stroes ESG et al. High-density lipoprotein particle size and concentration and coronary risk. Ann Intern Med 2009; 150: 84–93. 19153411 10.7326/0003-4819-150-2-200901200-00006

[bib44] Otvos JD, Collins D, Freedman DS, Shalaurova I, Schaefer EJ, McNamara JR et al. Low-density lipoprotein and high-density lipoprotein particle subclasses predict coronary events and are favorably changed by Gemfibrozil therapy in the veterans affairs high-density lipoprotein intervention trial. Circulation 2006; 113: 1556–1563. 16534013 10.1161/CIRCULATIONAHA.105.565135

[bib45] Esteve E, Ricart W, Fernández-Real JM. Dyslipidemia and inflammation: an evolutionary conserved mechanism. Clin Nutr 2005; 24: 16–31. 15681098 10.1016/j.clnu.2004.08.004

[bib46] Kelly CCJ, Lyall H, Petrie JR, Gould GW, Connell JMC, Sattar N. Low grade chronic inflammation in women with polycystic ovarian syndrome. J Clin Endocrinol Metab 2001; 86: 2453–2455. 11397838 10.1210/jcem.86.6.7580

[bib47] Meyer C, McGrath BP, Teede HJ. Overweight women with polycystic ovary syndrome have evidence of subclinical cardiovascular disease. J Clin Endocrinol Metab 2005; 90: 5711–5716. 16046590 10.1210/jc.2005-0011

[bib48] Elbers JM, Asscheman H, Seidell JC, Megens JA, Gooren LJ. Long-term testosterone administration increases visceral fat in female to male transsexuals 1. J Clin Endocrinol Metab 1997; 82: 2044–2047. 9215270 10.1210/jcem.82.7.4078

[bib49] Polderman KH, Gooren L, Asscheman H, Bakker A, Heine RJ. Induction of insulin resistance by androgens and estrogens. J Clin Endocrinol Metab 1994; 79: 265–271. 8027240 10.1210/jcem.79.1.8027240

[bib50] Diamond MP, Grainger D, Diamond MC, Sherwin RS, DeFronzo RA. Effects of methyltestosterone on insulin secretion and sensitivity in women 1. J Clin Endocrinol Metab 1998; 83: 4420–4425. 9851788 10.1210/jcem.83.12.5333

[bib51] Coviello AD, Legro RS, Dunaif A. Adolescent girls with polycystic ovary syndrome have an increased risk of the metabolic syndrome associated with increasing androgen levels independent of obesity and insulin resistance. J Clin Endocrinol Metab 2006; 91: 492–497. 16249280 10.1210/jc.2005-1666

[bib52] Wang X, Smith GI, Patterson BW, Reeds DN, Kampelman J, Magkos F et al. Testosterone increases the muscle protein synthesis rate but does not affect very-low-density lipoprotein metabolism in obese premenopausal women. Am J Physiol Endocrinol Metab 2012; 302: E740–E746. 22252942 10.1152/ajpendo.00533.2011 PMC3311295

[bib53] Colhoun HM, Otvos JD, Rubens MB, Taskinen MR, Underwood SR, Fuller JH. Lipoprotein subclasses and particle sizes and their relationship with coronary artery calcification in men and women with and without type 1 diabetes. Diabetes 2002; 51: 1949–1956. 12031985 10.2337/diabetes.51.6.1949

[bib54] Liepa GU, Sengupta A, Karsies D. Polycystic ovary syndrome (PCOS) and other androgen excess–related conditions: can changes in dietary intake make a difference? Nutr Clin Pract 2008; 23: 63–71. 18203965 10.1177/011542650802300163

[bib55] Dumesic DA, Akopians AL, Madrigal VK, Ramirez E, Margolis DJ, Sarma MK et al. Hyperandrogenism accompanies increased intra-abdominal fat storage in normal weight polycystic ovary syndrome women. J Clin Endocrinol Metab 2016; 101: 4178–4188. 27571186 10.1210/jc.2016-2586 PMC5095243

[bib56] Tonolo G, Ciccarese M, Brizzi P, Milia S, Dessole S, Puddu L et al. Cyclical variation of plasma lipids, apolipoproteins, and lipoprotein(a) during menstrual cycle of normal women. Am J Physiol 1995; 269: E1101–E1105. 8572203 10.1152/ajpendo.1995.269.6.E1101

[bib57] Mora S, Buring JE, Ridker PM, Cui Y. Association of high-density lipoprotein cholesterol with incident cardiovascular events in women, by low-density lipoprotein cholesterol and apolipoprotein B100 levelsA Cohort Study. Ann Intern Med 2011; 155: 742–750. 22147713 10.1059/0003-4819-155-11-201112060-00006 PMC3233986

