# Supplementary material for: Metabolic profiling of polycystic ovary syndrome reveals interactions with abdominal obesity
Source: Int J Obes (Lond). 2017 Jun 27;41(9):1331–40. doi: 10.1038/ijo.2017.126 (PMC5578435; doi:10.1038/ijo.2017.126)
Supplement: Supplementary Material [file ijo2017126x1.docx]

# SUPPLEMENTARY INFORMATION

# Metabolic Profiling of Polycystic Ovary Syndrome

**Authors**

Alexessander Couto Alves^1,*^, Beatriz Valcarcel^1,*^, Ville-Peteri Makinen^1^, Laure Morin-Papunen^2^, Sylvain Sebert^3,4^, Antti J Kangas^6^, Pasi Soininen^6,7^, Shikta Das^1^, Maria De Iorio^5^, Lachlan Coin^1^, Mika Ala Korpela^6,7,8^, Marjo-Riitta Jarvelin^1,3,4,10,#^, Stephen Franks^9#^

**Table of Contents**

SUPPLEMENTARY METHODS 2

SUPPLEMENTARY TABLES 3

# Supplementary methods

**Assessment of confounding factors**

We explored the impact of potential confounders; smoking, alcohol and socio-economic status by checking their association with PCOS and metabolites. The final assessment was based on a relative change in the regression coefficient after adding a potential confounder as a covariate to the model. A potential confounder was considered negligible on a specific association if, after including it in the model, the relative change in the regression coefficient was < 0.1 (i.e.10%). The effect of the confounder on the overall metabolic profile was considered negligible if for > 95% of the metabolic variables, the regression coefficient relative change was < 0.1.

# Supplementary Tables

Supplementary Table 1. Quantified metabolome-wide metabolites with their units. Measurements missing units, e.g. ratios, are unitless.

| Type | Abbreviation | Names | Unit |
| --- | --- | --- | --- |
| Albumin, lipoprotein subclasses and derived measures | Alb | Albumin | signal area |
|  | XXL_VLDL_PL | Phospholipids in chylomicrons and extremely large VLDL | mmol/l |
|  | XXL_VLDL_L | Total lipids in chylomicrons and extremely large VLDL | mmol/l |
|  | XXL_VLDL_P | Concentration of chylomicrons and extremely large VLDL particles | mol/l |
|  | XL_VLDL_PL | Phospholipids in very large VLDL | mmol/l |
|  | XL_VLDL_TG | Triglycerides in very large VLDL | mmol/l |
|  | XL_VLDL_L | Total lipids in very large VLDL | mmol/l |
|  | XL_VLDL_P | Concentration of very large VLDL particles | mol/l |
|  | L_VLDL_C | Total cholesterol in large VLDL | mmol/l |
|  | L_VLDL_FC | Free cholesterol in large VLDL | mmol/l |
|  | L_VLDL_PL | Phospholipids in large VLDL | mmol/l |
|  | L_VLDL_TG | Triglycerides in large VLDL | mmol/l |
|  | L_VLDL_CE | Cholesterol esters in large VLDL | mmol/l |
|  | L_VLDL_L | Total lipids in large VLDL | mmol/l |
|  | L_VLDL_P | Concentration of large VLDL particles | mol/l |
|  | M_VLDL_C | Total cholesterol in medium VLDL | mmol/l |
|  | M_VLDL_FC | Free cholesterol in medium VLDL | mmol/l |
|  | M_VLDL_PL | Phospholipids in medium VLDL | mmol/l |
|  | M_VLDL_TG | Triglycerides in medium VLDL | mmol/l |
|  | M_VLDL_CE | Cholesterol esters in medium VLDL | mmol/l |
|  | M_VLDL_L | Total lipids in medium VLDL | mmol/l |
|  | M_VLDL_P | Concentration of medium VLDL particles | mol/l |
|  | S_VLDL_C | Total cholesterol in small VLDL | mmol/l |
|  | S_VLDL_FC | Free cholesterol in small VLDL | mmol/l |
|  | S_VLDL_PL | Phospholipids in small VLDL | mmol/l |
|  | S_VLDL_TG | Triglycerides in small VLDL | mmol/l |
|  | S_VLDL_L | Total lipids in small VLDL | mmol/l |
|  | S_VLDL_P | Concentration of small VLDL particles | mol/l |
|  | XS_VLDL_PL | Phospholipids in very small VLDL | mmol/l |
|  | XS_VLDL_TG | Triglycerides in very small VLDL | mmol/l |
|  | XS_VLDL_L | Total lipids in very small VLDL | mmol/l |
|  | XS_VLDL_P | Concentration of very small VLDL particles | mol/l |
|  | IDL_FC | Free cholesterol in IDL | mmol/l |
|  | IDL_PL | Phospholipids in IDL | mmol/l |
|  | IDL_L | Total lipids in IDL | mmol/l |
|  | IDL_P | Concentration of IDL particles | mol/l |
|  | L_LDL_C | Total cholesterol in large LDL | mmol/l |
|  | L_LDL_FC | Free cholesterol in large LDL | mmol/l |
|  | L_LDL_PL | Phospholipids in large LDL | mmol/l |
|  | L_LDL_CE | Cholesterol esters in large LDL | mmol/l |
|  | L_LDL_L | Total lipids in large LDL | mmol/l |
|  | L_LDL_P | Concentration of large LDL particles | mol/l |
|  | M_LDL_C | Total cholesterol in medium LDL | mmol/l |
|  | M_LDL_PL | Phospholipids in medium LDL | mmol/l |
|  | M_LDL_CE | Cholesterol esters in medium LDL | mmol/l |
|  | M_LDL_L | Total lipids in medium LDL | mmol/l |
|  | M_LDL_P | Concentration of medium LDL particles | mol/l |
|  | S_LDL_C | Total cholesterol in small LDL | mmol/l |
|  | S_LDL_L | Total lipids in small LDL | mmol/l |
|  | S_LDL_P | Concentration of small LDL particles | mol/l |
|  | XL_HDL_C | Total cholesterol in very large HDL | mmol/l |
|  | XL_HDL_FC | Free cholesterol in very large HDL | mmol/l |
|  | XL_HDL_PL | Phospholipids in very large HDL | mmol/l |
|  | XL_HDL_TG | Triglycerides in very large HDL | mmol/l |
|  | XL_HDL_CE | Cholesterol esters in very large HDL | mmol/l |
|  | XL_HDL_L | Total lipids in very large HDL | mmol/l |
|  | XL_HDL_P | Concentration of very large HDL particles | mol/l |
|  | L_HDL_C | Total cholesterol in large HDL | mmol/l |
|  | L_HDL_FC | Free cholesterol in large HDL | mmol/l |
|  | L_HDL_PL | Phospholipids in large HDL | mmol/l |
|  | L_HDL_CE | Cholesterol esters in large HDL | mmol/l |
|  | L_HDL_L | Total lipids in large HDL | mmol/l |
|  | L_HDL_P | Concentration of large HDL particles | mol/l |
|  | M_HDL_C | Total cholesterol in medium HDL | mmol/l |
|  | M_HDL_FC | Free cholesterol in medium HDL | mmol/l |
|  | M_HDL_PL | Phospholipids in medium HDL | mmol/l |
|  | M_HDL_CE | Cholesterol esters in medium HDL | mmol/l |
|  | M_HDL_L | Total lipids in medium HDL | mmol/l |
|  | M_HDL_P | Concentration of medium HDL particles | mol/l |
|  | S_HDL_TG | Triglycerides in small HDL | mmol/l |
|  | S_HDL_L | Total lipids in small HDL | mmol/l |
|  | S_HDL_P | Concentration of small HDL particles | mol/l |
|  | XXL_VLDL_TG | Triglycerides in chylomicrons and extremely large VLDL | mmol/l |
|  | VLDL_TG | Triglycerides in VLDL | mmol/l |
|  | IDL_TG | Triglycerides in IDL | mmol/l |
|  | IDL_C | Total cholesterol in IDL | mmol/l |
|  | LDL_C | Total cholesterol in LDL | mmol/l |
|  | HDL_C | Total cholesterol in HDL | mmol/l |
|  | Serum_TG | Serum total triglycerides | mmol/l |
|  | Serum_C | Serum total cholesterol | mmol/l |
|  | VLDL_D | Mean diameter for VLDL particles | nm |
|  | LDL_D | Mean diameter for LDL particles | nm |
|  | HDL_D | Mean diameter for HDL particles | nm |
|  | VLDL_TG_eFR | Triglycerides in VLDL (Lipido) | mmol/l |
|  | IDL_C_eFR | Total cholesterol in IDL (Lipido) | mmol/l |
|  | LDL_C_eFR | Total cholesterol in LDL (Lipido) | mmol/l |
|  | HDL2_C | Total cholesterol in HDL2 (Lipido) | mmol/l |
|  | ApoA1 | Apolipoprotein A-I (Lipido) | g/l |
|  | ApoB | Apolipoprotein B (Lipido) | g/l |
|  | ApoBtoApoA1 | Apolipoprotein B by apolipoprotein A-I (Lipido) |  |
|  | HDL3_C | Total cholesterol in HDL3 (Lipido) | mmol/l |
| Amino acids and other low-molecular-weight metabolites (analysed here using intensity data) | bOHBut | 3-hydroxybutyrate | mmol/l |
|  | Ace | Acetate | mmol/l |
|  | AcAce | Acetoacetate | mmol/l |
|  | Ala | Alanine | mmol/l |
|  | MobCH2 | CH2 groups of mobile lipids |  |
|  | MobCH3 | CH3 groups of mobile lipids |  |
|  | Cit | Citrate | mmol/l |
|  | Crea | Creatinine | mmol/l |
|  | MobCH | Double bond protons of mobile lipids |  |
|  | Glc | Glucose | mmol/l |
|  | Gln | Glutamine | mmol/l |
|  | Glol | Glycerol | mmol/l |
|  | Gp | Glycoprotein acetyls, mainly a1-acid glycoprotein | mmol/l |
|  | His | Histidine | mmol/l |
|  | Ile | Isoleucine | mmol/l |
|  | Lac | Lactate | mmol/l |
|  | Leu | Leucine | mmol/l |
|  | Phe | Phenylalanine | mmol/l |
|  | Pyr | Pyruvate | mmol/l |
|  | Tyr | Tyrosine | mmol/l |
|  | Urea | Urea |  |
|  | Val | Valine | mmol/l |
| Serum lipid extracts | TotC | total cholesterol | mmol/l |
|  | EstC | esterified cholesterol | mmol/l |
|  | FreeC | free cholesterol | mmol/l |
|  | FAw3 | omega-3 fatty acids | mmol/l |
|  | FAw67 | omega-6 and -7 fatty acids | mmol/l |
|  | FAw9S | omega-9 and saturated fatty acids | mmol/l |
|  | TotFA | total fatty acids | mmol/l |
|  | LA | 18:2, linoleic acid (LA) | mmol/l |
|  | otPUFA | other polyunsaturated fatty acids than 18:2 | *** |
|  | DHA | 22:6, docosahexaenoic acid (DHA) | mmol/l |
|  | MUFA | total triglycerides | mmol/l |
|  | TotPG | total phosphoglycerides | mmol/l |
|  | PC | phosphatidylcholine and other cholines | mmol/l |
|  | SM | sphingomyelins | mmol/l |
|  | TotPCHOL | total cholines (and other N-trimethyl compounds) | mmol/l |
|  | FAw3toFA | ratio of omega-3 fatty acids to total fatty acids | % |
|  | FAw67toFA | ratio of omega-6/7 fatty acids to total fatty acids | % |
|  | FAw9StoFA | ratio of omega-9 and saturated fatty acids to total fatty acids | % |
|  | CH2inFA | average number of methylene groups in a fatty acid chain | - |
|  | TGtoPG | ratio of triglycerides to phosphoglycerides | - |
|  | CH2toDB | average number of methylene groups per a double bond | - |
|  | DBinFA | average number of double bonds in a fatty acid chain | - |
|  | BIStoDB | ratio of bisallylic groups to double bonds | - |
|  | BIStoFA | ratio of bisallylic groups to total fatty acids | - |
|  | FALen | description of average fatty acid chain length, not actual carbon number | - |

Supplementary Table 2. Confounder analysis show that >95% of the PCOS associations with lipoproteins, low molecular weight metabolites and lipids were not significantly affected (<10% of their original regression coefficient) by adding smoking, alcohol or socio-economic status as covariates. This suggests that these variables have a negligible effect on the large majority of metabolite associations (P<0.05), thus a reduced model adjusting for WC was used in this study.

| Confounder | The relative change in the metabolic associations with PCOS* at 5%, quartiles and 95% level of overall metabolic variables$ | | | | |
| --- | --- | --- | --- | --- | --- |
|  | 5% | 25% | 50% | 75% | 95% |
| Alcohol | 0.001 | 0.01 | 0.02 | 0.03 | 0.06 |
| Smoking | 0.03 | 0.05 | 0.06 | 0.06 | 0.08^#^ |
| Socio-economic status | 0.008 | 0.02 | 0.03 | 0.03 | 0.05 |

* Relative change of the regression coefficient is defined as the absolute difference on betas before and after adjustment divided by the beta before adjustment.

$ See methods above

^#^ Maximum relative change observed was 0.088.

Supplementary Table 3. Lipoprotein associations with PCOS adjusted for waist circumference (associations with p≤0.01 shown, n_cases_=145, n_control_=687, Odds Ratio, OR by SD increase in metabolite value).

|  | Standardized coefficients | | | | Model fit | |
| --- | --- | --- | --- | --- | --- | --- |
|  |  | 95% C.I. | |  |  |  |
| Metabolites standardized to unit variance | OR | Lower | Upper | P | Rsq | P |
| Triglycerides in small VLDL | 2.23 | 1.35 | 3.63 | **0.002** | 0.07 | 1.60E-08 |
| Cholesterol esters in large VLDL | 2.10 | 1.32 | 3.35 | **0.002** | 0.07 | 1.60E-08 |
| Total lipids in chylomicrons and extremely large VLDL | 2.08 | 1.32 | 3.35 | **0.002** | 0.07 | 1.30E-08 |
| Triglycerides in medium VLDL | 2.10 | 1.31 | 3.39 | **0.002** | 0.07 | 1.80E-08 |
| Concentration of medium VLDL particles | 2.10 | 1.31 | 3.35 | 0.002 | 0.07 | 2.00E-08 |
| Total lipids in medium VLDL | 2.10 | 1.31 | 3.39 | 0.002 | 0.07 | 2.00E-08 |
| Total cholesterol in large VLDL | 2.01 | 1.27 | 3.22 | 0.003 | 0.07 | 2.60E-08 |
| Free cholesterol in medium VLDL | 2.05 | 1.27 | 3.29 | 0.003 | 0.07 | 2.80E-08 |
| Concentration of very large VLDL particles | 1.99 | 1.27 | 3.22 | 0.003 | 0.07 | 2.40E-08 |
| Phospholipids in medium VLDL | 2.03 | 1.27 | 3.29 | 0.003 | 0.07 | 3.00E-08 |
| Total lipids in large VLDL | 1.99 | 1.26 | 3.19 | 0.003 | 0.07 | 2.80E-08 |
| Triglycerides in large VLDL | 1.99 | 1.26 | 3.19 | 0.004 | 0.07 | 2.80E-08 |
| Concentration of large VLDL particles | 1.97 | 1.26 | 3.16 | 0.004 | 0.07 | 3.00E-08 |
| Total cholesterol in medium VLDL | 2.01 | 1.25 | 3.25 | 0.004 | 0.07 | 3.50E-08 |
| Total lipids in very large VLDL | 1.95 | 1.25 | 3.13 | 0.004 | 0.07 | 3.20E-08 |
| Triglycerides in very large VLDL | 1.95 | 1.25 | 3.16 | 0.004 | 0.07 | 3.10E-08 |
| Phospholipids in chylomicrons and extremely large VLDL | 1.92 | 1.23 | 3.06 | 0.005 | 0.07 | 3.30E-08 |
| Free cholesterol in large VLDL | 1.92 | 1.22 | 3.06 | 0.01 | 0.07 | 4.40E-08 |
| Concentration of small VLDL particles | 2.01 | 1.22 | 3.29 | 0.01 | 0.06 | 5.00E-08 |
| Apolipoprotein B by apolipoprotein A I (Lipido) | 2.05 | 1.22 | 3.42 | 0.01 | 0.07 | 7.70E-08 |
| Cholesterol esters in medium VLDL | 1.93 | 1.20 | 3.13 | 0.01 | 0.06 | 5.70E-08 |
| Phospholipids in large VLDL | 1.88 | 1.19 | 3.00 | 0.01 | 0.06 | 5.70E-08 |
| Phospholipids in very large VLDL | 1.86 | 1.19 | 2.97 | 0.01 | 0.06 | 5.70E-08 |
| Phospholipids in large HDL | 0.47 | 0.26 | 0.81 | 0.01 | 0.06 | 5.10E-08 |
| Total lipids in small VLDL | 1.90 | 1.15 | 3.10 | 0.01 | 0.06 | 8.90E-08 |
| Concentration of large HDL particles | 0.49 | 0.28 | 0.85 | 0.01 | 0.06 | 7.80E-08 |
| Free cholesterol in large HDL | 0.49 | 0.28 | 0.85 | 0.01 | 0.06 | 8.20E-08 |
| Total lipids in large HDL | 0.50 | 0.28 | 0.86 | 0.01 | 0.06 | 8.70E-08 |
| Concentration of chylomicrons and extremely large VLDL | 1.72 | 1.11 | 2.69 | 0.02 | 0.06 | 1.20E-07 |

Supplementary Table 4. Lipids associations with PCOS adjusted for waist circumference (n_cases_=145, n_control_=687, Odds Ratio, OR by SD increase in metabolite value).

|  | Standardized odds ratio | | | | Model goodness of fit | |
| --- | --- | --- | --- | --- | --- | --- |
|  |  | 95% C.I. | |  |  |  |
| Metabolites standardized to unit variance | OR | Lower | Upper | P | Rsq | P |
| Ratio of triglycerides to phosphoglycerides | 2.08 | 1.27 | 3.39 | 0.003 | 0.07 | 3.00E-08 |
| Ratio of bisallylic groups to double bonds | 0.50 | 0.30 | 0.83 | 0.01 | 0.06 | 5.10E-08 |
| Ratio of bisallylic groups to total fatty acids | 0.53 | 0.32 | 0.88 | 0.01 | 0.06 | 9.40E-08 |
| Ratio of omega 9 and saturated fatty acids to total fatty acids | 1.80 | 1.11 | 2.97 | 0.02 | 0.06 | 1.40E-07 |
| Average number of double bonds in a fatty acid chain | 0.58 | 0.35 | 0.94 | 0.03 | 0.06 | 1.90E-07 |
| Average number of methylene groups per a double bond | 1.67 | 1.03 | 2.66 | 0.04 | 0.06 | 2.30E-07 |
| Monounsaturated fatty acids | 1.62 | 1.00 | 2.59 | 0.05 | 0.06 | 3.10E-07 |
| Ratio of omega 6 fatty acids to total fatty acids | 0.63 | 0.38 | 1.02 | 0.06 | 0.06 | 3.80E-07 |
| Omega 9 and saturated fatty acids | 1.49 | 0.92 | 2.39 | 0.1 | 0.06 | 5.70E-07 |
| Ratio of omega 3 fatty acids to total fatty acids | 0.66 | 0.38 | 1.08 | 0.11 | 0.06 | 5.80E-07 |
| Average fatty acid chain length | 0.73 | 0.46 | 1.19 | 0.2 | 0.05 | 9.30E-07 |
| Total fatty acids | 1.34 | 0.84 | 2.14 | 0.22 | 0.05 | 1.10E-06 |
| Average number of methylene groups in a fatty acid chain | 1.32 | 0.83 | 2.14 | 0.25 | 0.05 | 1.10E-06 |
| phosphatidylcholine and other cholines | 0.76 | 0.45 | 1.22 | 0.26 | 0.05 | 1.10E-06 |
| Free cholesterol | 1.16 | 0.72 | 1.84 | 0.54 | 0.05 | 1.60E-06 |
| omega 6 fatty acids | 1.09 | 0.67 | 1.73 | 0.72 | 0.05 | 2.10E-06 |
| Omega 3 fatty acids | 0.92 | 0.56 | 1.48 | 0.73 | 0.05 | 2.00E-06 |
| Sphingomyelins | 0.96 | 0.59 | 1.54 | 0.86 | 0.05 | 2.00E-06 |
| Linoleic acid | 1.03 | 0.63 | 1.65 | 0.91 | 0.05 | 2.00E-06 |
| Docosahexaenoic acid | 0.97 | 0.60 | 1.55 | 0.91 | 0.05 | 1.90E-06 |
| Total polyunsaturated fattyacids than linoleic acid ratio | 0.98 | 0.60 | 1.58 | 0.94 | 0.05 | 2.10E-06 |
| esterified cholesterol | 0.98 | 0.61 | 1.57 | 0.95 | 0.05 | 1.90E-06 |
| Total phosphoglycerides | 1.00 | 0.62 | 1.60 | 0.99 | 0.05 | 2.20E-06 |

Supplementary Table 5. Low molecular weight metabolites association with PCOS adjusted for waist circumference (n_cases_=145, n_control_=687, Odds Ratio, OR by SD increase in metabolite value).

|  | Standardized odds ratio | | | | Model goodness of fit | |
| --- | --- | --- | --- | --- | --- | --- |
|  |  | 95% C.I. | |  |  |  |
| Metabolites standardized to unit variance | OR | Lower | Upper | P | Rsq | P |
| CH3 groups of mobile lips | 1.80 | 1.14 | 2.83 | 0.01 | 0.06 | 6.30E-08 |
| Urea | 0.56 | 0.34 | 0.91 | 0.02 | 0.06 | 1.10E-07 |
| Acetate | 0.60 | 0.35 | 1.00 | 0.06 | 0.06 | 2.30E-07 |
| Isoleucine | 1.46 | 0.90 | 2.36 | 0.12 | 0.06 | 4.80E-07 |
| CH2 groups of mobile lips | 1.36 | 0.85 | 2.14 | 0.19 | 0.05 | 6.70E-07 |
| Double bond protons of mobile lips | 1.36 | 0.85 | 2.14 | 0.19 | 0.05 | 6.80E-07 |
| Histine | 1.28 | 0.80 | 1.99 | 0.29 | 0.05 | 9.10E-07 |
| Creatinine | 0.78 | 0.47 | 1.27 | 0.32 | 0.05 | 9.50E-07 |
| Leucine | 1.23 | 0.75 | 2.01 | 0.41 | 0.05 | 1.10E-06 |
| Glycoprotein acetyls mainly a1 a glycoprotein | 0.84 | 0.49 | 1.38 | 0.48 | 0.05 | 1.20E-06 |
| Lactate | 0.85 | 0.52 | 1.36 | 0.51 | 0.05 | 1.10E-06 |
| Glycerol | 1.15 | 0.71 | 1.84 | 0.55 | 0.05 | 1.30E-06 |
| Glucose | 0.88 | 0.53 | 1.40 | 0.59 | 0.05 | 1.40E-06 |
| Alanine | 1.11 | 0.68 | 1.77 | 0.66 | 0.05 | 1.40E-06 |
| Valine | 0.90 | 0.54 | 1.45 | 0.67 | 0.05 | 1.40E-06 |
| Acetoacetate | 0.90 | 0.54 | 1.45 | 0.70 | 0.05 | 1.60E-06 |
| Citrate | 1.08 | 0.66 | 1.77 | 0.76 | 0.06 | 1.70E-07 |
| Hydroxybutyrate | 0.92 | 0.49 | 1.43 | 0.77 | 0.05 | 1.50E-06 |
| Pyruvate | 1.06 | 0.66 | 1.68 | 0.81 | 0.05 | 1.20E-06 |
| Phenylalanine | 0.94 | 0.55 | 1.57 | 0.83 | 0.05 | 1.50E-06 |
| Glutamine | 1.04 | 0.63 | 1.68 | 0.88 | 0.07 | 1.10E-07 |
| Tyrosine | 0.98 | 0.59 | 1.57 | 0.93 | 0.05 | 2.00E-06 |

Supplementary Table 6. Mean concentration of serum lipids in PCOS and control women stratified by waist circumference (WC) cut-offs for clinical action.

|  | WC <87 cm | | | 87 cm < WC < 98 cm | | | WC >98 cm | | | Interaction |
| --- | --- | --- | --- | --- | --- | --- | --- | --- | --- | --- |
|  | Cases | Controls |  | Cases | Controls |  | Cases | Controls |  | 2-Way ANOVA |
| Metabolite (units) | (n=93) | (n=562) | p-value | (n=26) | (n=82) | p-value | (n=26) | (n=46) | p-value | p-value |
| Total cholines (mmol/l) | 2.2 | 2.17 | 0.57 | 2.35 | 2.32 | 0.76 | 2.06 | 2.4 | **0.002** | 0.02 |
| total phosphoglycerides (mmol/l) | 0.86 | 0.85 | 0.56 | 0.93 | 0.92 | 0.87 | 0.81 | 0.98 | 0.003 | 0.03 |
| total cholesterol (mmol/l) | 5.02 | 4.9 | 0.31 | 5.56 | 5.44 | 0.66 | 4.88 | 5.63 | 0.004 | 0.01 |
| esterified cholesterol (mmol/l) | 3.71 | 3.6 | 0.24 | 4.08 | 4.06 | 0.92 | 3.58 | 4.17 | 0.005 | 0.01 |
| sphingomyelins (mmol/l) | 0.35 | 0.34 | 0.45 | 0.4 | 0.38 | 0.46 | 0.35 | 0.4 | 0.005 | 0.01 |
| phosphatidylcholine and other cholines (mmol/l) | 1.91 | 1.9 | 0.82 | 2.04 | 2.04 | 1 | 1.8 | 2.13 | 0.005 | 0.06 |
| ω6 and ω7 fatty acids (mmol/l) | 3.52 | 3.41 | 0.18 | 3.91 | 3.88 | 0.83 | 3.55 | 4.02 | 0.019 | 0.04 |
| ω6-linoleic acid (mmol/l) | 3.21 | 3.12 | 0.27 | 3.45 | 3.53 | 0.62 | 3.06 | 3.46 | 0.02 | 0.06 |
| other polyunsaturated fatty acids than linoleic acid (mmol/l) | 1.91 | 1.87 | 0.46 | 2.15 | 2.03 | 0.28 | 1.95 | 2.23 | 0.02 | 0.04 |
| free cholesterol (mmol/l) | 1.31 | 1.3 | 0.76 | 1.48 | 1.38 | 0.19 | 1.32 | 1.46 | 0.04 | 0.07 |
| ω3-docosahexaenoic acid (mmol/l) | 0.12 | 0.12 | 0.63 | 0.13 | 0.11 | 0.25 | 0.1 | 0.13 | 0.05 | 0.06 |
| triglycerides to phosphoglycerides ratio | 0.92 | 0.85 | 0.13 | 1.39 | 1.06 | **0.03** | 1.51 | 1.23 | 0.06 | 0.14 |
| ω3 fatty acid (mmol/l) | 0.25 | 0.26 | 0.51 | 0.31 | 0.27 | 0.1 | 0.25 | 0.3 | 0.12 | 0.09 |
| ratio of omega-9 and saturated fatty acids to total fatty acids | 57.26 | 57.1 | 0.58 | 59.86 | 57.48 | 0.004 | 60.45 | 59.1 | 0.13 | 0.02 |
| total fatty acids (mmol/l) | 8.83 | 8.56 | 0.18 | 10.67 | 9.77 | 0.12 | 9.75 | 10.71 | 0.15 | 0.12 |
| ratio of omega-6/7 fatty acids to total fatty acids | 39.94 | 39.91 | 0.93 | 37.19 | 39.76 | **0.002** | 36.91 | 38.12 | 0.18 | 0.01 |
| average number of methylene groups per a double bond | 7.6 | 7.56 | 0.48 | 8 | 7.73 | 0.08 | 8.13 | 7.96 | 0.33 | 0.23 |
| omega-9 and saturated fatty acids (mmol/l) | 5.06 | 4.89 | 0.17 | 6.45 | 5.63 | 0.04 | 5.95 | 6.39 | 0.34 | 0.13 |
| average number of double bonds in a fatty acid chain | 1.48 | 1.49 | 0.74 | 1.41 | 1.48 | 0.01 | 1.4 | 1.43 | 0.35 | 0.07 |
| ratio of bisallylic groups to total fatty acids | 0.79 | 0.8 | 0.13 | 0.74 | 0.78 | 0.06 | 0.73 | 0.75 | 0.36 | 0.49 |
| ratio of bisallylic groups to double bonds | 0.53 | 0.54 | 0.02 | 0.52 | 0.53 | 0.43 | 0.52 | 0.52 | 0.45 | 0.9 |
| ratio of omega-3 fatty acids to total fatty acids | 2.8 | 2.99 | 0.13 | 2.95 | 2.76 | 0.33 | 2.63 | 2.79 | 0.52 | 0.25 |
| average fatty acid chain length | 20.35 | 20.34 | 0.89 | 20.1 | 20.46 | 0.04 | 20.19 | 20.28 | 0.66 | 0.17 |
| total triglycerides (mmol/l) | 0.78 | 0.7 | 0.08 | 1.27 | 0.97 | 0.05 | 1.24 | 1.21 | 0.84 | 0.35 |
| average number of methylene groups in a fatty acid chain | 11.23 | 11.21 | 0.77 | 11.2 | 11.34 | 0.24 | 11.32 | 11.32 | 0.99 | 0.52 |

Supplementary Table 7. Mean concentration of serum lipoproteins in PCOS and control women stratified by waist circumference (WC) cut-offs for clinical action (only significant results shown). The p-value of the 2-way ANOVA of the interaction between WC and PCOS status on metabolite levels is significant (P<0.002) for Albumin and suggestive for Apolipoprotein A I (P<0.004).

|  | WC<87 | | | 87<WC<98 | | | WC>98 | | | Interaction |
| --- | --- | --- | --- | --- | --- | --- | --- | --- | --- | --- |
|  | Cases | Controls |  | Cases | Controls |  | Cases | Controls |  | 2-Way ANOVA |
| Metabolite | (n=93) | (n=562) | p-value | (n=26) | (n=82) | p-value | (n=26) | (n=46) | p-value | p-value |
| Phospholipids in large HDL (mmol/l) | 4.40E-01 | 4.70E-01 | 0.2 | 3.50E-01 | 4.20E-01 | 0.03 | 2.30E-01 | 3.70E-01 | **3.40E-05** | 0.041 |
| Concentration of large HDL particles (mol/l) | 1.20E-06 | 1.30E-06 | 0.25 | 9.50E-07 | 1.10E-06 | 0.03 | 6.10E-07 | 1.00E-06 | **3.50E-05** | 0.038 |
| Total lipids in large HDL (mmol/l) | 9.70E-01 | 1.00E+00 | 0.27 | 7.50E-01 | 9.10E-01 | 0.03 | 4.80E-01 | 8.00E-01 | **5.00E-05** | 0.042 |
| Free cholesterol in large HDL (mmol/l) | 1.00E-01 | 1.10E-01 | 0.22 | 7.70E-02 | 9.60E-02 | 0.03 | 4.50E-02 | 8.10E-02 | **7.00E-05** | 0.063 |
| *Apolipoprotein A I Lipido (g/l)* | *1.70E+00* | *1.70E+00* | *0.85* | *1.70E+00* | *1.70E+00* | *0.54* | *1.50E+00* | *1.70E+00* | ***9.60E-05*** | *0.003* |
| Total cholesterol in HDL (mmol/l) | 1.70E+00 | 1.80E+00 | 0.51 | 1.60E+00 | 1.70E+00 | 0.04 | 1.30E+00 | 1.60E+00 | **1.00E-04** | 0.02 |
| **Albumin** | 1.00E-01 | 1.00E-01 | 0.95 | 1.10E-01 | 1.10E-01 | 0.6 | 9.20E-02 | 1.00E-01 | **1.40E-04** | **0.002** |
| Total cholesterol in HDL2 Lipido (mmol/l) | 1.20E+00 | 1.30E+00 | 0.51 | 1.00E+00 | 1.20E+00 | 0.03 | 7.80E-01 | 1.10E+00 | **1.70E-04** | 0.038 |
| Total cholesterol in large HDL (mmol/l) | 5.10E-01 | 5.30E-01 | 0.37 | 3.80E-01 | 4.70E-01 | 0.02 | 2.50E-01 | 4.10E-01 | **2.10E-04** | 0.056 |
| Free cholesterol in medium HDL (mmol/l) | 8.80E-02 | 9.10E-02 | 0.53 | 8.60E-02 | 9.20E-02 | 0.33 | 6.30E-02 | 8.90E-02 | **2.30E-04** | 0.024 |
| Mean diameter for HDL particles (nm) | 1.00E+01 | 1.00E+01 | 0.31 | 9.90E+00 | 1.00E+01 | 0.07 | 9.70E+00 | 9.90E+00 | **3.00E-04** | 0.072 |
| Cholesterol esters in large HDL (mmol/l) | 4.10E-01 | 4.20E-01 | 0.44 | 3.00E-01 | 3.70E-01 | 0.02 | 2.10E-01 | 3.30E-01 | **3.10E-04** | 0.051 |
| Total cholesterol in medium HDL (mmol/l) | 5.00E-01 | 5.10E-01 | 0.59 | 4.70E-01 | 5.00E-01 | 0.26 | 3.60E-01 | 4.80E-01 | **5.30E-04** | 0.03 |
| Cholesterol esters in medium HDL (mmol/l) | 4.10E-01 | 4.20E-01 | 0.61 | 3.80E-01 | 4.10E-01 | 0.25 | 3.00E-01 | 4.00E-01 | **7.10E-04** | 0.033 |
|  |  |  |  |  |  |  |  |  |  |  |
| Total lipids in medium HDL (mmol/l) | 9.30E-01 | 9.50E-01 | 0.53 | 9.10E-01 | 9.50E-01 | 0.4 | 7.20E-01 | 9.30E-01 | **7.60E-04** | 0.041 |
| Concentration of medium HDL particles (mol/l) | 1.70E-06 | 1.70E-06 | 0.52 | 1.70E-06 | 1.70E-06 | 0.49 | 1.30E-06 | 1.70E-06 | **9.80E-04** | 0.048 |
| Phospholipids in medium HDL (mmol/l) | 4.10E-01 | 4.20E-01 | 0.46 | 4.10E-01 | 4.20E-01 | 0.45 | 3.30E-01 | 4.20E-01 | **1.20E-03** | 0.064 |
| Serum total cholesterol (mmol/l) | 5.00E+00 | 4.90E+00 | 0.29 | 5.60E+00 | 5.40E+00 | 0.66 | 4.90E+00 | 5.60E+00 | 4.30E-03 | 0.012 |
| Phospholipids in very large HDL (mmol/l) | 2.60E-01 | 2.70E-01 | 0.38 | 2.00E-01 | 2.30E-01 | 0.16 | 1.60E-01 | 2.20E-01 | 5.20E-03 | 0.311 |
| Total lipids in small HDL (mmol/l) | 1.10E+00 | 1.10E+00 | 0.87 | 1.20E+00 | 1.20E+00 | 0.93 | 1.10E+00 | 1.20E+00 | 8.20E-03 | 0.059 |

Supplementary Table 8. Testosterone (nmol/l) association with serum lipids in women with abdominal obesity (WC>98). Model fit to cases and controls separately: testosterone = metabolite + WC.

|  | PCOS (n=26) | | | CONTROLS (n=46) | | | Comparison |
| --- | --- | --- | --- | --- | --- | --- | --- |
| Metabolite | β | se | p-value | β | se | p-value | p-value |
| average number of methylene groups per a double bond (mmol/l) | 1.04 | 0.21 | **3.6E-07** | -0.02 | 0.09 | 0.4 | **2.8E-06** |
| ratio of triglycerides to phosphoglycerides | 1.41 | 0.3 | **9.8E-07** | -0.11 | 0.11 | 0.16 | **1.5E-06** |
| ratio of bisallylic groups to total fatty acids | -6.3 | 1.47 | **9.5E-06** | 0.56 | 0.56 | 0.16 | **1.4E-05** |
| ratio of bisallylic groups to double bonds | -20.38 | 5.25 | **5.1E-05** | 2.38 | 1.89 | 0.1 | **4.5E-05** |
| total triglycerides (mmol/l) | 1.1 | 0.3 | **1.1E-04** | -0.01 | 0.1 | 0.45 | **3.9E-04** |
| average number of double bonds in a fatty acid chain | -4.91 | 1.37 | **1.6E-04** | 0.41 | 0.49 | 0.2 | **2.4E-04** |
| omega-9 and saturated fatty acids (mmol/l) | 0.17 | 0.05 | **4.4E-04** | -0.02 | 0.02 | 0.1 | **3.6E-04** |
| ratio of omega-9 and saturated fatty acids to total fatty acids | 0.33 | 0.11 | **1.9E-03** | 0 | 0.03 | 0.45 | 5.5E-03 |
| ratio of omega-6/7 fatty acids to total fatty acids | -0.16 | 0.06 | 3.8E-03 | 0.02 | 0.02 | 0.11 | 3.9E-03 |
| ratio of omega-3 fatty acids to total fatty acids | -0.67 | 0.26 | 5.7E-03 | 0 | 0.04 | 0.5 | 1.2E-02 |
| total fatty acids (mmol/l) | 0.2 | 0.09 | 1.6E-02 | 0.01 | 0.02 | 0.35 | 4.4E-02 |
| 22:6, docosahexaenoic acid (DHA) (mmol/l) | -11.38 | 6.01 | 2.9E-02 | 0.02 | 1 | 0.49 | 6.1E-02 |
| description of average fatty acid chain length, not actual carbon number | -0.41 | 0.26 | 5.6E-02 | 0.05 | 0.06 | 0.2 | 8.2E-02 |
| free cholesterol (mmol/l) | -1.01 | 1.22 | 2.0E-01 | 0.09 | 0.14 | 0.27 | 3.7E-01 |
| other polyunsaturated fatty acids than 18:2 | -0.51 | 0.63 | 2.1E-01 | 0.07 | 0.09 | 0.22 | 3.6E-01 |
| omega-3 fatty acids (mmol/l) | -2.26 | 2.88 | 2.2E-01 | 0.01 | 0.38 | 0.48 | 4.3E-01 |
| phosphatidylcholine and other cholines (mmol/l) | 0.4 | 0.63 | 2.6E-01 | 0.12 | 0.1 | 0.12 | 6.6E-01 |
| total cholesterol (mmol/l) | -0.15 | 0.29 | 3.0E-01 | 0.03 | 0.04 | 0.21 | 5.2E-01 |
| omega-6 and -7 fatty acids (mmol/l) | 0.18 | 0.41 | 3.3E-01 | 0.05 | 0.06 | 0.18 | 7.6E-01 |
| esterified cholesterol (mmol/l) | -0.15 | 0.36 | 3.4E-01 | 0.05 | 0.06 | 0.2 | 5.8E-01 |
| total phosphoglycerides (mmol/l) | 0.55 | 1.53 | 3.6E-01 | 0.22 | 0.21 | 0.14 | 8.3E-01 |
| total cholines (and other N-trimethyl compounds) (mmol/l) | 0.29 | 0.83 | 3.6E-01 | 0.08 | 0.09 | 0.19 | 8.1E-01 |
| Sphingomyelins (mmol/l) | -1.7 | 5.17 | 3.7E-01 | 0.41 | 0.59 | 0.24 | 6.8E-01 |
| average number of methylene groups in a fatty acid chain | -0.12 | 0.47 | 4.0E-01 | 0.05 | 0.08 | 0.25 | 7.2E-01 |
| 18:2, linoleic acid (LA) (mmol/l) | 0.02 | 0.47 | 4.8E-01 | 0.07 | 0.07 | 0.17 | 9.2E-01 |

Supplementary Table 9. Testosterone (nmol/l) association with serum lipoproteins for women with abdominal obesity (WC>98cm). Model fit to cases and controls separately: testosterone = metabolite + WC. Only associations with P<0.05 shown.

|  | PCOS |  |  | CONTROLS | |  | Comparison |
| --- | --- | --- | --- | --- | --- | --- | --- |
| Metabolite | β | se | p-value | β | se | p-value | p-value |
| Triglycerides in very large VLDL (mmol/l) | 2.3E+01 | 4.5E+00 | **1.0E-07** | -6.1E-01 | 1.3E+00 | 3.2E-01 | **3.0E-07** |
| Total lipids in very large VLDL (mmol/l) | 1.4E+01 | 2.9E+00 | **3.2E-07** | -4.3E-01 | 8.2E-01 | 3.0E-01 | **8.2E-07** |
| Phospholipids in chylomicrons and extremely large VLDL (mmol/l) | 2.4E+02 | 4.9E+01 | **5.6E-07** | -1.0E+01 | 2.0E+01 | 3.0E-01 | **2.6E-06** |
| Phospholipids in very large VLDL (mmol/l) | 7.2E+01 | 1.5E+01 | **6.4E-07** | -2.6E+00 | 4.5E+00 | 2.8E-01 | **1.6E-06** |
| Triglycerides in large VLDL (mmol/l) | 9.2E+00 | 2.0E+00 | **2.4E-06** | -1.7E-01 | 3.9E-01 | 3.3E-01 | **4.8E-06** |
| Total lipids in chylomicrons and extremely large VLDL (mmol/l) | 2.8E+01 | 6.1E+00 | **2.5E-06** | -1.4E+00 | 2.3E+00 | 2.6E-01 | **6.9E-06** |
| Triglycerides in chylomicrons and extremely large VLDL (mmol/l) | 3.9E+01 | 8.7E+00 | **2.9E-06** | -2.0E+00 | 3.3E+00 | 2.7E-01 | **8.5E-06** |
| Concentration of chylomicrons and extremely large VLDL particles (mol/l) | 6.1E+09 | 1.3E+09 | **3.0E-06** | -1.9E+08 | 5.0E+08 | 3.5E-01 | **1.2E-05** |
| Total lipids in large VLDL (mmol/l) | 5.3E+00 | 1.2E+00 | **3.3E-06** | -1.2E-01 | 2.3E-01 | 3.0E-01 | **5.9E-06** |
| Concentration of very large VLDL particles (mol/l) | 1.4E+09 | 3.0E+08 | **4.0E-06** | -3.7E+07 | 8.4E+07 | 3.3E-01 | **9.8E-06** |
| Free cholesterol in large VLDL (mmol/l) | 4.2E+01 | 9.8E+00 | **1.0E-05** | -9.4E-01 | 2.0E+00 | 3.2E-01 | **2.0E-05** |
| Phospholipids in large VLDL (mmol/l) | 2.8E+01 | 6.7E+00 | **1.4E-05** | -7.2E-01 | 1.2E+00 | 2.8E-01 | **2.4E-05** |
| Concentration of large VLDL particles | 3.2E+08 | 7.9E+07 | **2.8E-05** | -6.7E+06 | 1.4E+07 | 3.1E-01 | **5.0E-05** |
| Total cholesterol in large VLDL (mmol/l) | 2.1E+01 | 5.5E+00 | **5.0E-05** | -7.2E-01 | 9.9E-01 | 2.3E-01 | **7.6E-05** |
| Triglycerides in VLDL (mmol/l) | 3.2E+00 | 8.4E-01 | **6.0E-05** | -6.8E-02 | 1.1E-01 | 2.7E-01 | **9.9E-05** |
| Mean diameter for VLDL particles (nm) | 7.3E-01 | 2.0E-01 | **1.7E-04** | -1.1E-02 | 4.2E-02 | 4.0E-01 | **3.8E-04** |
| Triglycerides in medium VLDL (mmol/l) | 8.0E+00 | 2.2E+00 | **1.8E-04** | -1.5E-01 | 2.9E-01 | 3.1E-01 | **3.2E-04** |
| Concentration of medium VLDL particles | 1.6E+08 | 4.6E+07 | **3.4E-04** | -3.1E+06 | 5.6E+06 | 2.9E-01 | **5.8E-04** |
| Mean diameter for LDL particles (nm) | -3.4E+00 | 1.0E+00 | **3.9E-04** | -3.1E-02 | 3.4E-01 | 4.6E-01 | **1.6E-03** |
| Cholesterol esters in large VLDL (mmol/l) | 4.1E+01 | 1.2E+01 | **4.0E-04** | -1.9E+00 | 2.0E+00 | 1.7E-01 | **5.3E-04** |
| Total lipids in medium VLDL (mmol/l) | 4.3E+00 | 1.3E+00 | **6.8E-04** | -9.2E-02 | 1.6E-01 | 2.8E-01 | **1.2E-03** |
| Triglycerides in VLDL Lipido (mmol/l) | 2.8E+00 | 9.2E-01 | **1.4E-03** | -1.0E-01 | 1.2E-01 | 2.0E-01 | 2.1E-03 |
| Free cholesterol in medium VLDL (mmol/l) | 2.9E+01 | 1.0E+01 | 2.8E-03 | -6.8E-01 | 1.3E+00 | 2.9E-01 | 4.8E-03 |
| Phospholipids in medium VLDL (mmol/l) | 1.9E+01 | 7.2E+00 | 4.6E-03 | -5.3E-01 | 8.2E-01 | 2.6E-01 | 7.8E-03 |
| Serum total triglycerides (mmol/l) | 1.7E+00 | 7.1E-01 | 9.4E-03 | -5.6E-02 | 8.9E-02 | 2.6E-01 | 1.6E-02 |
| Triglycerides in small VLDL (mmol/l) | 9.7E+00 | 5.1E+00 | 3.0E-02 | -3.6E-01 | 4.7E-01 | 2.2E-01 | 5.1E-02 |
| Total cholesterol in medium VLDL (mmol/l) | 8.3E+00 | 5.0E+00 | 4.8E-02 | -3.9E-01 | 6.1E-01 | 2.6E-01 | 8.4E-02 |

Supplementary Table 10. Testosterone (nmol/l) association with low molecular weight metabolites for women with abdominal obesity (WC>98cm). Model fit to cases and controls separately: testosterone = metabolite + WC.

|  | PCOS |  |  | CONTROLS | |  | Comparison |
| --- | --- | --- | --- | --- | --- | --- | --- |
| Metabolite | β | se | p-value | β | se | p-value | p-value |
| CH2.groups.of.mobile.lips | 1.5E-07 | 4.5E-08 | **3.1E-04** | 3.2E-09 | 1.5E-08 | 4.1E-01 | **1.5E-03** |
| Glycerol | 3.9E-05 | 1.7E-05 | 1.1E-02 | 8.0E-07 | 3.9E-06 | 4.2E-01 | 2.8E-02 |
| Alanine | 2.3E-05 | 1.0E-05 | 1.1E-02 | 1.6E-07 | 2.0E-06 | 4.7E-01 | 2.6E-02 |
| Glycoprotein.acetyls..mainly.a1.ac.glycoprotein | 8.0E-06 | 3.7E-06 | 1.5E-02 | 6.1E-07 | 6.8E-07 | 1.9E-01 | 4.8E-02 |
| CH3.groups.of.mobile.lips | 4.8E-07 | 2.4E-07 | 2.1E-02 | 2.8E-08 | 6.4E-08 | 3.3E-01 | 6.4E-02 |
| Urea | -6.4E-05 | 3.2E-05 | 2.3E-02 | 9.0E-07 | 7.9E-06 | 4.5E-01 | 4.9E-02 |
| Double.bond.protons.of.mobile.lips | 2.2E-06 | 1.1E-06 | 2.7E-02 | 1.1E-07 | 3.0E-07 | 3.6E-01 | 7.9E-02 |
| Tyrosine | 1.0E-04 | 6.3E-05 | 4.9E-02 | -9.6E-06 | 1.2E-05 | 2.0E-01 | 7.6E-02 |
| Glucose | 2.9E-06 | 2.1E-06 | 8.6E-02 | -4.5E-07 | 4.7E-07 | 1.6E-01 | 1.2E-01 |
| Leucine | 5.0E-05 | 4.6E-05 | 1.3E-01 | 6.1E-06 | 1.0E-05 | 2.8E-01 | 3.4E-01 |
| Pyruvate | 3.1E-05 | 3.2E-05 | 1.7E-01 | -3.8E-06 | 5.7E-06 | 2.5E-01 | 2.9E-01 |
| Histine | 7.2E-05 | 7.6E-05 | 1.7E-01 | 9.0E-06 | 1.4E-05 | 2.6E-01 | 4.1E-01 |
| Acetoacetate | 1.2E-05 | 1.7E-05 | 2.4E-01 | -1.3E-06 | 3.0E-06 | 3.3E-01 | 4.5E-01 |
| Isoleucine | 3.9E-05 | 6.4E-05 | 2.7E-01 | -2.3E-06 | 1.2E-05 | 4.2E-01 | 5.3E-01 |
| Phenylalanine | 2.6E-05 | 5.6E-05 | 3.2E-01 | 8.6E-07 | 1.4E-05 | 4.7E-01 | 6.6E-01 |
| X3.hydroxybutyrate | 2.7E-06 | 6.0E-06 | 3.3E-01 | -6.5E-07 | 1.3E-06 | 3.1E-01 | 5.9E-01 |
| Lactate | -1.4E-06 | 3.3E-06 | 3.3E-01 | 2.2E-07 | 4.3E-07 | 3.0E-01 | 6.2E-01 |
| Citrate | -1.7E-05 | 4.8E-05 | 3.6E-01 | -6.6E-06 | 7.1E-06 | 1.8E-01 | 8.3E-01 |
| Acetate | -2.7E-05 | 9.8E-05 | 3.9E-01 | -3.7E-06 | 2.1E-05 | 4.3E-01 | 8.2E-01 |
| Creatinine | -2.7E-05 | 1.0E-04 | 4.0E-01 | 9.7E-07 | 1.7E-05 | 4.8E-01 | 7.9E-01 |
| Valine | -3.1E-06 | 2.4E-05 | 4.5E-01 | -5.6E-08 | 3.6E-06 | 4.9E-01 | 9.0E-01 |
| Glutamine | 1.5E-06 | 1.2E-05 | 4.5E-01 | 5.2E-07 | 1.8E-06 | 3.9E-01 | 9.4E-01 |
